# Rearrangement Events on Circular Genomes

**DOI:** 10.1007/s11538-023-01209-5

**Published:** 2023-09-25

**Authors:** Joshua Stevenson, Venta Terauds, Jeremy Sumner

**Affiliations:** 1 University of Tasmania, Hobart, Australia; 2 University of South Australia, Adelaide, Australia

**Keywords:** Genome rearrangements, Phylogenetics, Combinatorics

## Abstract

Early literature on genome rearrangement modelling views the problem of computing evolutionary distances as an inherently combinatorial one. In particular, attention is given to estimating distances using the minimum number of events required to transform one genome into another. In hindsight, this approach is analogous to early methods for inferring phylogenetic trees from DNA sequences such as maximum parsimony—both are motivated by the principle that the true distance minimises evolutionary change, and both are effective if this principle is a true reflection of reality. Recent literature considers genome rearrangement under statistical models, continuing this parallel with DNA-based methods, with the goal of using model-based methods (for example maximum likelihood techniques) to compute distance estimates that incorporate the large number of rearrangement paths that can transform one genome into another. Crucially, this approach requires one to decide upon a set of feasible rearrangement events and, in this paper, we focus on characterising well-motivated models for signed, uni-chromosomal circular genomes, where the number of regions remains fixed. Since rearrangements are often mathematically described using permutations, we isolate the sets of permutations representing rearrangements that are biologically reasonable in this context, for example inversions and transpositions. We provide precise mathematical expressions for these rearrangements, and then describe them in terms of the set of cuts made in the genome when they are applied. We directly compare cuts to breakpoints, and use this concept to count the distinct rearrangement actions which apply a given number of cuts. Finally, we provide some examples of rearrangement models, and include a discussion of some questions that arise when defining plausible models.

## Introduction

The goal of genome rearrangement modelling is to construct phylogenetic trees from a given set of genomes which share segments of DNA, but differ in the arrangement of these segments (a segment may be reversed, for example). These segments of DNA may be contiguous collections of genes (sometimes referred to as synteny blocks) or genes themselves. We will refer to these conserved segments of DNA simply as *regions*. Genome rearrangements are events which affect segments of one or more regions of DNA within a genome: physically breaking the genome in one or more places and then reconnecting in some way. Often, phylogenetic trees are constructed by first estimating the evolutionary distance between every pair of genomes under consideration, and then using a distance-based tree reconstruction method to obtain a phylogeny. Evolutionary distance estimates are obtained by considering the possible genome rearrangements which can be applied to transform a given genome into another.

The theory of genome rearrangements has developed in a similar way to a number of other areas in phylogenetics. For both the reconstruction of phylogenetic trees, and the more specific problem of computing evolutionary distance between genomes, the most obvious solution is to determine the minimum ‘amount’ of evolution required, in accordance with the Occam’s razor principle. In the case of tree reconstruction, this leads to ‘maximum parsimony’ and related approaches—methods that the general phylogenetics literature has largely moved away from over the last few decades, due to problems with bias in certain situations, as was demonstrated by Felsenstein (1973). Similarly, the straight-forward combinatorial approaches to estimating evolutionary distance between genomes (namely, ‘minimal distance’ under various sets of allowed rearrangements) have been successful in many ways, yielding fast algorithms for computing particular distances (Hannenhalli and Pevzner 1996; Bader et al. 2001), but have had limitations in terms of flexibility of both the models and the very definition of rearrangement distance. Identifying biologically realistic sets of rearrangements and determining their associated probabilities is still a work in progress. Further, some models may be more appropriate than others for particular groups of organisms. For these reasons, model inflexibility represents a disadvantage of these combinatorial algorithms.

In contrast to combinatorial approaches to computing genome rearrangement distances, which generally assume a fixed set of possible events occurring with equal probability and aim to identify the shortest possible path, *model-based* approaches are those that incorporate a probability distribution of possible events, which can be parameterised and altered without the need for new algorithms. Model-based methods can also have outputs other than the shortest possible paths, such as the mean first passage time (MFPT) (Francis and Wynn 2020; Terauds et al. 2021) or maximum likelihood estimate (MLE) of elapsed time between genomes (Serdoz et al. 2017). An inherent difficulty in genome rearrangement modelling is the computational complexity arising from the number of genomes, which is factorial in the number of regions. For example, in this paper we examine signed, circular, single-chromosomal genomes, and there are $2^{n-1}(n-1)!$ such genomes with *n* regions. For combinatorial approaches that aim to estimate distances as the shortest number of discrete rearrangements, only a small subset of the possible paths between any given pair of genomes is considered. However, for model-based distance estimates such as the MLE, all possible paths between pairs of genomes are considered.

Despite their inherent factorial complexity, it is becoming increasingly clear that the next phase of development of genome rearrangement theory will rely on these model-based approaches, together with a flexible array of available distance measures and tree reconstruction methods [see Serdoz et al. (2017), Terauds and Sumner (2018), Egri-Nagy et al. (2013), Bhatia et al. (2018), Francis (2013)]. In our view, developing effective model-based methods for computing genome rearrangement distances will require formalising existing concepts and specifically expanding upon them from an algebraic perspective. Bhatia et al. (2018) make an excellent contribution toward this goal, clarifying a number of concepts used in the genome rearrangement literature. We aim to expand upon this contribution through the ideas and results we present here. With a focus on rearrangements, we connect numerous competing standards in the literature, centring on one particular representation of genomes that we argue is a natural choice for model-based approaches. Further, we provide an overview of the precise algebraic operations required to translate between different algebraic representations of genomes and rearrangements as detailed in Bhatia et al. (2018).

In this paper, we identify and precisely describe, in algebraic terms, the biologically significant classes of rearrangements—including the most prevalent cases which have appeared in the literature—and discuss these in the context of model-based methods. In Sect. 2.1, we give an overview of the various existing representations of genomes and rearrangements as permutations, and motivate our choice of representation, which we use for the remainder of the paper. Section 2.2 focuses on symmetry: in particular, we discuss how the symmetries of circular genomes and rearrangements inform the algebraic objects we use to represent rearrangement events, as well as the way in which we define models. We subsequently discuss rearrangement models from a practical point of view in Sect. 3, giving examples to illustrate some important considerations for choosing rearrangement probabilities and estimating genome rearrangement distances. We introduce and formally define the ‘set of cuts’ required to perform a given rearrangement in Sect. 4, and use this idea to characterise and for the first time establish explicit expressions for the number of distinct rearrangement actions which are precisely the biologically reasonable ones. We also discuss the relationship between this idea and the important and well-studied concept of a ‘breakpoint’. The paper concludes with a summary including a brief discussion of possible future work.

## Background

### Genomes and rearrangements as permutations

Approaches to genome rearrangement modelling can differ greatly depending on the particular types of genomes in question. We will focus on modelling uni-chromosomal circular genomes with a fixed number of oriented regions, and ‘genome’ will refer to a genome under these assumptions unless otherwise stated. Genomes of this type are found in bacteria (with some exceptions); see, for example Thanbichler and Shapiro (2006). This choice excludes some rearrangement events (namely fissions, fusions, duplications and deletions) which change the number of chromosomes or the number of regions, however this still leaves our set of possible rearrangement events much larger than the set of all inversions, for example, which is a model commonly studied in the literature (Hannenhalli and Pevzner 1996; Caprara 1997; Berard et al. 2007; Bafna and Pevzner 1996; Bader et al. 2001; Baudet et al. 2015; Lin and Moret 2008).

Whether the associated algebraic structures are utilised or not, permutations are commonly used to represent genomes in the literature and have been since as early as 1982 (Watterson et al. 1982). Even when given a particular set of constraints—such as the assumption that genomes are circular—there are numerous choices which need to be made in terms of the way we represent genomes and rearrangement events as permutations. Bhatia et al. (2018) categorise these choices into two main paradigms, namely the ‘position’ and ‘content’ paradigms. The key difference is that in the position paradigm, the locations of regions in the genome are encoded via a fixed position labelling, whereas in the content paradigm, we only keep track of each region’s location in relation to other regions. To complicate matters, even after choosing a paradigm, there remain multiple choices of mathematical representation. We presently explain one particular way to describe genomes using the position paradigm, and then explore other representations, including ones falling under the content paradigm.

To represent a genome in the position paradigm, we first label the *n* different genomic regions with the set of integers $[n]:=\{ 1,\ldots ,n \}$. We then describe the position and orientation of each region as a map from the set of regions to the set of signed positions $[\hspace{0.55542pt}\overline{\hspace{-0.55542pt}n\hspace{-0.55542pt}}\hspace{0.55542pt}]:= \{1,\ldots , n, \hspace{0.55542pt}\overline{\hspace{-0.55542pt}1\hspace{-0.55542pt}}\hspace{0.55542pt},\ldots \hspace{0.55542pt}\overline{\hspace{-0.55542pt}n\hspace{-0.55542pt}}\hspace{0.55542pt}\}$, where the bar above a position label indicates a reversed region. For example, consider a genome with 5 regions represented by the map $$\sigma = \left[ {\begin{matrix} 1 &{} 2 &{} 3 &{} 4 &{} 5 \\ \hspace{0.55542pt}\overline{\hspace{-0.55542pt}1\hspace{-0.55542pt}}\hspace{0.55542pt}&{} 4 &{} 3 &{} \hspace{0.55542pt}\overline{\hspace{-0.55542pt}5\hspace{-0.55542pt}}\hspace{0.55542pt}&{} \hspace{0.55542pt}\overline{\hspace{-0.55542pt}2\hspace{-0.55542pt}}\hspace{0.55542pt}\end{matrix}}\right] $$, where the top row represents the pre-image (regions) and the bottom row represents the image (signed positions). Often the top row is omitted, which is unambiguous when the region labels are ordered as they are here. Note that in this form, the map $\sigma$ is not a bijection, because its pre-image is [*n*]. To remedy this, we extend the domain of $\sigma$ to all of $[\hspace{0.55542pt}\overline{\hspace{-0.55542pt}n\hspace{-0.55542pt}}\hspace{0.55542pt}]$ via the rule

1 $$\begin{aligned} \sigma (\hspace{0.55542pt}\overline{\hspace{-0.55542pt}\,i\,\hspace{-0.55542pt}}\hspace{0.55542pt}) := \hspace{0.55542pt}\overline{\hspace{-0.55542pt}\sigma (i)\hspace{-0.55542pt}}\hspace{0.55542pt}, \end{aligned}$$

for all $i\in [n]$. The extended map $\sigma$ is a bijection and hence a permutation on the set $[\hspace{0.55542pt}\overline{\hspace{-0.55542pt}n\hspace{-0.55542pt}}\hspace{0.55542pt}]$. The subset of permutations on $[\hspace{0.55542pt}\overline{\hspace{-0.55542pt}n\hspace{-0.55542pt}}\hspace{0.55542pt}]$ which satisfy (1) forms a subgroup of $S_{[\hspace{0.55542pt}\overline{\hspace{-0.55542pt}n\hspace{-0.55542pt}}\hspace{0.55542pt}]}$ (the group of permutations on $[\hspace{0.55542pt}\overline{\hspace{-0.55542pt}n\hspace{-0.55542pt}}\hspace{0.55542pt}]$), namely the hyperoctahedral group, which we denote as ${{\,\mathrm{{\mathcal {H}}}\,}}_n$. This extension means we can write $\sigma$ simply as $$\left[ {\begin{matrix} 1 &{} 2 &{} 3 &{} 4 &{} 5 \\ \hspace{0.55542pt}\overline{\hspace{-0.55542pt}1\hspace{-0.55542pt}}\hspace{0.55542pt}&{} 4 &{} 3 &{} \hspace{0.55542pt}\overline{\hspace{-0.55542pt}5\hspace{-0.55542pt}}\hspace{0.55542pt}&{} \hspace{0.55542pt}\overline{\hspace{-0.55542pt}2\hspace{-0.55542pt}}\hspace{0.55542pt}\end{matrix}}\right] $$, or in one-row notation as $[\hspace{0.55542pt}\overline{\hspace{-0.55542pt}1\hspace{-0.55542pt}}\hspace{0.55542pt}\ 4\ 3\ \hspace{0.55542pt}\overline{\hspace{-0.55542pt}5\hspace{-0.55542pt}}\hspace{0.55542pt}\ \hspace{0.55542pt}\overline{\hspace{-0.55542pt}2\hspace{-0.55542pt}}\hspace{0.55542pt}]$, remembering that its action on the remaining elements of $[\hspace{0.55542pt}\overline{\hspace{-0.55542pt}n\hspace{-0.55542pt}}\hspace{0.55542pt}]$ is given by Eq. (1). We have a natural embedding of ${\mathcal {S}}_n$ as a subgroup of ${{\,\mathrm{{\mathcal {H}}}\,}}_n$, obtained by extending each permutation in ${\mathcal {S}}_n$ via the above process. In this paradigm, rearrangements are also expressed as elements of the hyperoctahedral group, and interpreted as maps from the set $[\hspace{0.55542pt}\overline{\hspace{-0.55542pt}n\hspace{-0.55542pt}}\hspace{0.55542pt}]$ of signed positions to itself.

We now convert the genome $$\sigma = \left[ {\begin{matrix} 1 &{} 2 &{} 3 &{} 4 &{} 5 \\ \hspace{0.55542pt}\overline{\hspace{-0.55542pt}1\hspace{-0.55542pt}}\hspace{0.55542pt}&{} 4 &{} 3 &{} \hspace{0.55542pt}\overline{\hspace{-0.55542pt}5\hspace{-0.55542pt}}\hspace{0.55542pt}&{} \hspace{0.55542pt}\overline{\hspace{-0.55542pt}2\hspace{-0.55542pt}}\hspace{0.55542pt}\end{matrix}}\right] $$ to illustrate some of the other commonly-used permutation representations of genomes. To aid our explanation, consider the two permutations $c=(1\ldots n)(\hspace{0.55542pt}\overline{\hspace{-0.55542pt}n\hspace{-0.55542pt}}\hspace{0.55542pt}\ldots \hspace{0.55542pt}\overline{\hspace{-0.55542pt}1\hspace{-0.55542pt}}\hspace{0.55542pt})$ and $r=(1\hspace{0.55542pt}\overline{\hspace{-0.55542pt}1\hspace{-0.55542pt}}\hspace{0.55542pt})\ldots (n\hspace{0.55542pt}\overline{\hspace{-0.55542pt}n\hspace{-0.55542pt}}\hspace{0.55542pt})$ in $S_{[\hspace{0.55542pt}\overline{\hspace{-0.55542pt}n\hspace{-0.55542pt}}\hspace{0.55542pt}]}$, written in cycle notation (i.e, *c* maps $1\mapsto 2,\ 2\mapsto 3,\ \ldots ,\ n\mapsto 1,\ \hspace{0.55542pt}\overline{\hspace{-0.55542pt}1\hspace{-0.55542pt}}\hspace{0.55542pt}\mapsto \hspace{0.55542pt}\overline{\hspace{-0.55542pt}n\hspace{-0.55542pt}}\hspace{0.55542pt},\ \hspace{0.55542pt}\overline{\hspace{-0.55542pt}2\hspace{-0.55542pt}}\hspace{0.55542pt}\mapsto \hspace{0.55542pt}\overline{\hspace{-0.55542pt}1\hspace{-0.55542pt}}\hspace{0.55542pt}$,..., $\hspace{0.55542pt}\overline{\hspace{-0.55542pt}n\hspace{-0.55542pt}}\hspace{0.55542pt}\mapsto \hspace{0.55542pt}\overline{\hspace{-0.55542pt}n\!-\!1\hspace{-0.55542pt}}\hspace{0.55542pt}$, and *r* maps $1\leftrightarrow \hspace{0.55542pt}\overline{\hspace{-0.55542pt}1\hspace{-0.55542pt}}\hspace{0.55542pt}$,..., $n\leftrightarrow \hspace{0.55542pt}\overline{\hspace{-0.55542pt}n\hspace{-0.55542pt}}\hspace{0.55542pt}$). Note that *c* is not an element of ${{\,\mathrm{{\mathcal {H}}}\,}}_n$ because it does not satisfy equation (1), whereas *r* does and hence $r\in {\mathcal {H}}_n$. Using the permutations *c* and *r*, we can convert $\sigma$ into three further representations of the same genome. These are detailed in Table 1.

**Fig. 1:**
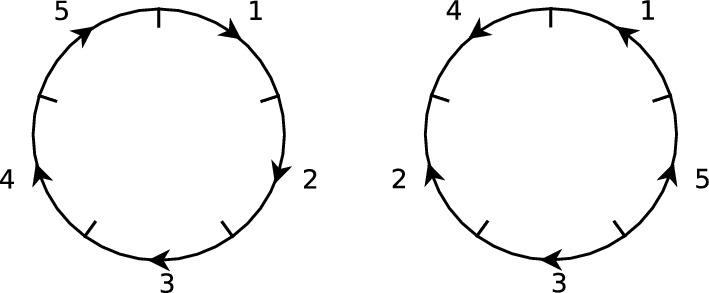
On the left is a drawing of the 5-region genome represented by the identity permutation $$\left[ {\begin{matrix} 1 &{} 2 &{} 3 &{} 4 &{} 5 \\ 1 &{} 2 &{} 3 &{} 4 &{} 5 \end{matrix}}\right] $$, which we refer to as the *reference genome*. On the right is a drawing of the genome represented by $$\sigma = \left[ {\begin{matrix} 1 &{} 2 &{} 3 &{} 4 &{} 5 \\ \hspace{0.55542pt}\overline{\hspace{-0.55542pt}1\hspace{-0.55542pt}}\hspace{0.55542pt}&{} 4 &{} 3 &{} \hspace{0.55542pt}\overline{\hspace{-0.55542pt}5\hspace{-0.55542pt}}\hspace{0.55542pt}&{} \hspace{0.55542pt}\overline{\hspace{-0.55542pt}2\hspace{-0.55542pt}}\hspace{0.55542pt}\end{matrix}}\right] $$. Alternate representations are summarised in Table 1

**Table 1:** Converting a permutation $$\sigma = \left[ {\begin{matrix} 1 &{} 2 &{} 3 &{} 4 &{} 5 \\ \hspace{0.55542pt}\overline{\hspace{-0.55542pt}1\hspace{-0.55542pt}}\hspace{0.55542pt}&{} 4 &{} 3 &{} \hspace{0.55542pt}\overline{\hspace{-0.55542pt}5\hspace{-0.55542pt}}\hspace{0.55542pt}&{} \hspace{0.55542pt}\overline{\hspace{-0.55542pt}2\hspace{-0.55542pt}}\hspace{0.55542pt}\end{matrix}}\right] $$ (shown in Fig. 1) serving as a representation of the genome in the position paradigm (reg $\rightarrow$ pos) to various other paradigms

Expression	Paradigm	Version	Sign means	Example
$\sigma$	Position	Region $\rightarrow$ position	Orientation	$[\hspace{0.55542pt}\overline{\hspace{-0.55542pt}1\hspace{-0.55542pt}}\hspace{0.55542pt}\ 4\ 3\ \hspace{0.55542pt}\overline{\hspace{-0.55542pt}5\hspace{-0.55542pt}}\hspace{0.55542pt}\ \hspace{0.55542pt}\overline{\hspace{-0.55542pt}2\hspace{-0.55542pt}}\hspace{0.55542pt}]$
$\sigma ^{-1}$	Position	Position $\rightarrow$ region	Orientation	$[\hspace{0.55542pt}\overline{\hspace{-0.55542pt}1\hspace{-0.55542pt}}\hspace{0.55542pt}\ \hspace{0.55542pt}\overline{\hspace{-0.55542pt}5\hspace{-0.55542pt}}\hspace{0.55542pt}\ 3\ 2\ \hspace{0.55542pt}\overline{\hspace{-0.55542pt}4\hspace{-0.55542pt}}\hspace{0.55542pt}]$
$\sigma ^{-1}c\sigma$	Content	Cycles	Orientation	$(\hspace{0.55542pt}\overline{\hspace{-0.55542pt}1\hspace{-0.55542pt}}\hspace{0.55542pt}\hspace{0.55542pt}\overline{\hspace{-0.55542pt}5\hspace{-0.55542pt}}\hspace{0.55542pt}32\hspace{0.55542pt}\overline{\hspace{-0.55542pt}4\hspace{-0.55542pt}}\hspace{0.55542pt})(4\hspace{0.55542pt}\overline{\hspace{-0.55542pt}2\hspace{-0.55542pt}}\hspace{0.55542pt}\hspace{0.55542pt}\overline{\hspace{-0.55542pt}3\hspace{-0.55542pt}}\hspace{0.55542pt}51)$
$\sigma ^{-1}c\sigma r$	Content	Adjacencies	$\hspace{0.55542pt}\overline{\hspace{-0.55542pt}\text {Head}\hspace{-0.55542pt}}\hspace{0.55542pt}$/tail	$(1\hspace{0.55542pt}\overline{\hspace{-0.55542pt}5\hspace{-0.55542pt}}\hspace{0.55542pt})(53)(\hspace{0.55542pt}\overline{\hspace{-0.55542pt}3\hspace{-0.55542pt}}\hspace{0.55542pt}2)(\hspace{0.55542pt}\overline{\hspace{-0.55542pt}2\hspace{-0.55542pt}}\hspace{0.55542pt}\hspace{0.55542pt}\overline{\hspace{-0.55542pt}4\hspace{-0.55542pt}}\hspace{0.55542pt})(4\hspace{0.55542pt}\overline{\hspace{-0.55542pt}1\hspace{-0.55542pt}}\hspace{0.55542pt})$

Recall that since $n=5$ in this example, we have $c=(12345)(\hspace{0.55542pt}\overline{\hspace{-0.55542pt}5\hspace{-0.55542pt}}\hspace{0.55542pt}\hspace{0.55542pt}\overline{\hspace{-0.55542pt}4\hspace{-0.55542pt}}\hspace{0.55542pt}\hspace{0.55542pt}\overline{\hspace{-0.55542pt}3\hspace{-0.55542pt}}\hspace{0.55542pt}\hspace{0.55542pt}\overline{\hspace{-0.55542pt}2\hspace{-0.55542pt}}\hspace{0.55542pt}\hspace{0.55542pt}\overline{\hspace{-0.55542pt}1\hspace{-0.55542pt}}\hspace{0.55542pt})$ and $r=(1\hspace{0.55542pt}\overline{\hspace{-0.55542pt}1\hspace{-0.55542pt}}\hspace{0.55542pt})(2\hspace{0.55542pt}\overline{\hspace{-0.55542pt}2\hspace{-0.55542pt}}\hspace{0.55542pt})(3\hspace{0.55542pt}\overline{\hspace{-0.55542pt}3\hspace{-0.55542pt}}\hspace{0.55542pt})(4\hspace{0.55542pt}\overline{\hspace{-0.55542pt}4\hspace{-0.55542pt}}\hspace{0.55542pt})(5\hspace{0.55542pt}\overline{\hspace{-0.55542pt}5\hspace{-0.55542pt}}\hspace{0.55542pt})$

Note that each representation is defined based on the interpretation of the input and output of the permutation. The position paradigm representations are either maps from regions to positions or maps from positions to regions. The content paradigm representations are maps from each region to the following region (in a clockwise direction) or maps between heads and tails of regions (in this case, the sign of the region number indicates either a head or tail, rather than orientation of a region). Note that the examples in the final column of Table 1 are written in a notation chosen to most easily express the genome in the particular paradigm. It is also common to see permutations representing position paradigm genomes, for example, written in cycle notation as well.

Since the region and position labels are arbitrary, when one wishes to represent a collection of genomes as permutations, it is common to designate a particular genome as the *reference genome*, naming its regions clockwise with the integers 1 through *n*. In the position paradigm, a coinciding position labelling may be chosen and, accordingly, the reference genome can be represented by the identity permutation $[1\ 2\ \cdots \ n]$. In the content (cycles) paradigm, which is independent of position labels, the reference genome is represented simply by the permutation $c =(12\ldots n)(\hspace{0.55542pt}\overline{\hspace{-0.55542pt}n\hspace{-0.55542pt}}\hspace{0.55542pt}\ldots \hspace{0.55542pt}\overline{\hspace{-0.55542pt}2\hspace{-0.55542pt}}\hspace{0.55542pt}\hspace{0.55542pt}\overline{\hspace{-0.55542pt}1\hspace{-0.55542pt}}\hspace{0.55542pt})$. For an example see Fig. 1.

The expressions in the first column in Table 1 are concise because we started in the position paradigm. This paradigm contains the most information, in the sense that the particular orientation of a circular genome can be differentiated, since regions are positioned absolutely, rather than in a way that is relative to neighbouring regions, as is the case in the content paradigm. If we began with a genome in the content paradigm, obtaining the equivalent genome in the position paradigm is a less pleasant process. For example, to transform $\pi = (\hspace{0.55542pt}\overline{\hspace{-0.55542pt}1\hspace{-0.55542pt}}\hspace{0.55542pt}\hspace{0.55542pt}\overline{\hspace{-0.55542pt}5\hspace{-0.55542pt}}\hspace{0.55542pt}32\hspace{0.55542pt}\overline{\hspace{-0.55542pt}4\hspace{-0.55542pt}}\hspace{0.55542pt})(4\hspace{0.55542pt}\overline{\hspace{-0.55542pt}2\hspace{-0.55542pt}}\hspace{0.55542pt}\hspace{0.55542pt}\overline{\hspace{-0.55542pt}3\hspace{-0.55542pt}}\hspace{0.55542pt}51)$ (content/cycles paradigm) in Table 1 into $\sigma ^{-1} = [\hspace{0.55542pt}\overline{\hspace{-0.55542pt}1\hspace{-0.55542pt}}\hspace{0.55542pt}\ \hspace{0.55542pt}\overline{\hspace{-0.55542pt}5\hspace{-0.55542pt}}\hspace{0.55542pt}\ 3\ 2\ \hspace{0.55542pt}\overline{\hspace{-0.55542pt}4\hspace{-0.55542pt}}\hspace{0.55542pt}]$ (position paradigm, positions-to-regions), we must consider one of the cycles as the bottom row of a signed permutation. Of course, there are two cycles to choose from and each may be written in *n* different ways, due to dihedral symmetry of the genome. If we decide to choose the cycle containing ‘1’, we can write $\sigma ^{-1}$ down in a more systematic way, by setting $\sigma ^{-1}(1) = 1$, $\sigma ^{-1}(i) = \pi ^{i-1}(1)$ for $i\ne 1$ and then extending via $\sigma ^{-1}(\hspace{0.55542pt}\overline{\hspace{-0.55542pt}i\hspace{-0.55542pt}}\hspace{0.55542pt}) = \hspace{0.55542pt}\overline{\hspace{-0.55542pt}\sigma ^{-1}(i)\hspace{-0.55542pt}}\hspace{0.55542pt}$. Still, this is far from being as nice as the simple algebraic expressions in the first column of Table 1.

Paradigm choice also determines how rearrangements are represented and applied. For example, in the position paradigm where genomes are maps from regions to positions, rearrangements are thought of as maps from positions to positions. In this case, applying these rearrangements is done via composition. For example, if $\sigma$ represents a genome and $\alpha$ represents a rearrangement, then $\alpha \sigma$ represents a genome where the positions of the regions have been shuffled around by $\alpha$; region *i* is now in position $\alpha (\sigma (i))$. In the content paradigm, considering genomes to be products of two *n*-cycles (as in the third row of Table 1), rearrangements are maps from regions to regions, and are applied via conjugation. If $\sigma ^{-1}c\sigma$ represents a genome in the content paradigm, then $\sigma ^{-1}c\sigma (i)=j$ means that region *i* is followed by region *j*. For example, the rearrangement $\alpha = (jk)$ maps region *j* to region *k* and is applied via conjugation, and thus $\alpha ^{-1}\sigma ^{-1}c\sigma \alpha (i)$ will map *i* to *k*.

A clear benefit of the content paradigm is that we have a one-to-one correspondence with circular genomes, without needing to resort to cosets or formal sums of permutations, as is the case when working in the position paradigm (see Sect. 2.2 for details). An important disadvantage though, is that the outcome of ‘applying’ a particular rearrangement to a genome in the content paradigm depends on the genome itself. For example, Meidanis and Dias state: *“There is no way to define a class of permutations that will be ‘the reversals’, valid for all genomes”* (Meidanis and Dias 2000), referring to defining inversions as permutations to be applied by left multiplication in the content paradigm. This makes it difficult to algebraically define a model in which not all rearrangements are equally probable, although an appropriate group action can be defined by converting to the position paradigm, applying the rearrangement and then converting back. For example, the genome is written $\sigma c \sigma ^{-1}$, and the permutation $\alpha$ (representing a rearrangement in the position paradigm) is applied using the group action defined via $\sigma c \sigma ^{-1} \longmapsto \sigma \alpha c \alpha ^{-1} \sigma ^{-1}$, where $\sigma$ is a permutation representing a genome in the position paradigm. Under this group action, we can in fact write down a set of all permutations which represent reversals, for example.

Conversely, in the position paradigm, we can (in theory) define a set of rearrangements which performs a specific type of action (for example, inversions of two adjacent regions) and can be applied in the standard way. In Sect. 4, we define ‘*k*-cut’ rearrangements in the position paradigm. An important difference between our definition and the definition of a ‘*k*-break’ rearrangement in the content paradigm [algebraically, conjugation of a genome by a *k*-cycle (Feijao and Meidanis 2013; Bhatia et al. 2018)], is that the action of a *k*-cut rearrangement will always require exactly *k* cuts, whereas, the action of a *k*-break operation may differ depending on the genome it is being applied to. Even when the rearrangement does require *k* cuts, the distribution of cuts across the genome will depend on the genome itself. While this disadvantage makes representing genomes and rearrangements using the content paradigm for model-based approaches impractical, the content paradigm has provided the field with a number of important results and efficient algorithms. As described in Bhatia et al. (2018), moving between paradigms can provide additional understanding. In fact, in Sect. 4 we will use the permutation $c=(1\ldots n)(\hspace{0.55542pt}\overline{\hspace{-0.55542pt}n\hspace{-0.55542pt}}\hspace{0.55542pt}\ldots \hspace{0.55542pt}\overline{\hspace{-0.55542pt}1\hspace{-0.55542pt}}\hspace{0.55542pt})$ to aid our description of different classes of rearrangements.

### Circular symmetries and equivalent rearrangements

**Fig. 2:**
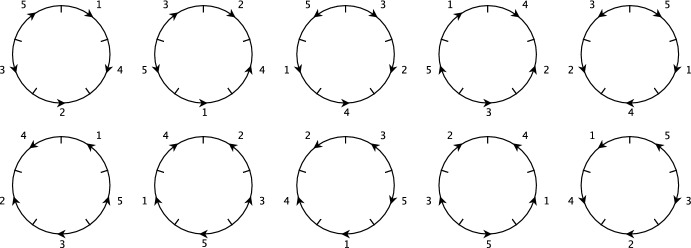
Diagrams representing each of the $2n=2(5)=10$ instances of the example genome ${\mathcal {D}}_n\sigma$ arising from its dihedral symmetries. The bottom left diagram matches the instance $\sigma = \left[ \hspace{0.55542pt}\overline{\hspace{-0.55542pt}1\hspace{-0.55542pt}}\hspace{0.55542pt}\ 4\ 3\ \hspace{0.55542pt}\overline{\hspace{-0.55542pt}5\hspace{-0.55542pt}}\hspace{0.55542pt}\ \hspace{0.55542pt}\overline{\hspace{-0.55542pt}2\hspace{-0.55542pt}}\hspace{0.55542pt}\right]$ (also shown in Fig. 1). Positions run clockwise starting from position 1 in the top-right of the circle

In Sect. 2.1, we discussed the fact that in the content paradigm, there is a one-to-one correspondence between genomes and valid permutations. In the position paradigm this is not the case, but can be easily corrected as we will see shortly. In the following, we consider circular genomes under the position paradigm (regions $\rightarrow$ positions). Whether regions are oriented or otherwise, circular genomes modelled with no distinguished positions [for example, an origin of replication, as considered by some authors (Darling et al. 2008; Egri-Nagy et al. 2013)] have dihedral symmetry; that is, circular genomes are physical objects that are unchanged by rotations and flips in physical space. Formally, they are invariant under the action of the dihedral group ${\mathcal {D}}_n$. Because of this, there are $|{\mathcal {D}}_n| = 2n$ distinct signed permutations corresponding to each physical genome. We refer to each of these permutations that represents a genome as an *instance* of the genome. For example, Fig. 2 shows several diagrams representing a particular genome, each portraying the same genome in a different physical orientation, and thus corresponding to a different signed permutation. To express this algebraically, we consider which rearrangements in ${{\,\mathrm{{\mathcal {H}}}\,}}_n$ leave the *genome* unchanged. In our formulation, a single position rotation of a genome is given by the rearrangement

$$\begin{aligned} r:= (123\ldots n)(\hspace{0.55542pt}\overline{\hspace{-0.55542pt}1\hspace{-0.55542pt}}\hspace{0.55542pt}\hspace{0.55542pt}\overline{\hspace{-0.55542pt}2\hspace{-0.55542pt}}\hspace{0.55542pt}\hspace{0.55542pt}\overline{\hspace{-0.55542pt}3\hspace{-0.55542pt}}\hspace{0.55542pt}\ldots \hspace{0.55542pt}\overline{\hspace{-0.55542pt}n\hspace{-0.55542pt}}\hspace{0.55542pt}) \in {{\,\mathrm{{\mathcal {H}}}\,}}_n. \end{aligned}$$

This is simply the analogous rotation in the dihedral group ${\mathcal {D}}_n$ extended to ${{\,\mathrm{{\mathcal {H}}}\,}}_n$. Reflections are more complicated, however, as we will see. For $n=5$, an example of a reflection is

$$\begin{aligned} f:= (2,\hspace{0.55542pt}\overline{\hspace{-0.55542pt}5\hspace{-0.55542pt}}\hspace{0.55542pt})(\hspace{0.55542pt}\overline{\hspace{-0.55542pt}2\hspace{-0.55542pt}}\hspace{0.55542pt},5)(3,\hspace{0.55542pt}\overline{\hspace{-0.55542pt}4\hspace{-0.55542pt}}\hspace{0.55542pt})(\hspace{0.55542pt}\overline{\hspace{-0.55542pt}3\hspace{-0.55542pt}}\hspace{0.55542pt},4)(1,\hspace{0.55542pt}\overline{\hspace{-0.55542pt}1\hspace{-0.55542pt}}\hspace{0.55542pt}) \in {{\,\mathrm{{\mathcal {H}}}\,}}_n. \end{aligned}$$

We cannot obtain this by taking the permutation $(2,5)(3,4) \in {\mathcal {D}}_5$ representing an unoriented reflection and simply extending to ${{\,\mathrm{{\mathcal {H}}}\,}}_5$ via Eq. (1), as one might first think. This is because for genomes with oriented regions, reflections change the orientation of the regions. Regardless, the group generated by the signed permutations *f* and *r* is precisely the set of rearrangements which leave a given genome unchanged, and is isomorphic to ${\mathcal {D}}_n$. This can be seen by considering the definition of the dihedral group involving two generators, $\left\langle {r}, {f} \mid {r}^{n}={f}^{2}=({fr})^{2}=e\right\rangle \cong {\mathcal {D}}_n$. Throughout the paper we will denote this copy of the dihedral group as ${\mathcal {D}}_n$.

Dihedral symmetry can be incorporated into calculations by considering genomes as cosets rather than individual permutations, as was introduced by Serdoz et al. (2017). In this case, each genome corresponds to a particular coset. For example, the genome corresponding to the permutation $\sigma$ is given by the coset ${\mathcal {D}}_5\sigma = \{ d\sigma : d \in {\mathcal {D}}_5 \}$, collecting all the reflections and rotations of $\sigma$, which we individually refer to as *instances* of the genome ${\mathcal {D}}_5\sigma$. While cosets are a natural way to factor out symmetries, we will see shortly that it is more appropriate to use a different set of mathematical objects to represent genomes and rearrangements.

Genome rearrangements can also exhibit symmetry. When we multiply a permutation ($\alpha : \textrm{positions} \rightarrow \textrm{positions}$) representing a rearrangement into an instance of a genome $d\sigma \in {\mathcal {D}}_n\sigma$, the instance $\alpha d \sigma$ that we get back might not represent the same genome as the result of $\alpha$ acting on some other instance, say $d_2\sigma \in {\mathcal {D}}_n\sigma$. We cannot simply choose another element of ${\mathcal {D}}_n \alpha$ instead to correct this. For example, the permutations $(1\hspace{0.55542pt}\overline{\hspace{-0.55542pt}1\hspace{-0.55542pt}}\hspace{0.55542pt})$ and $(1\hspace{0.55542pt}\overline{\hspace{-0.55542pt}1\hspace{-0.55542pt}}\hspace{0.55542pt})(12345)(\hspace{0.55542pt}\overline{\hspace{-0.55542pt}12345\hspace{-0.55542pt}}\hspace{0.55542pt})$, are both expressions showing the application of a rearrangement $(1\hspace{0.55542pt}\overline{\hspace{-0.55542pt}1\hspace{-0.55542pt}}\hspace{0.55542pt})$ to an instance of the reference genome (i.e., an element of ${\mathcal {D}}_n$), but the resulting permutations are not both instances of the same genome. For this reason, Terauds et al. (2021) consider formal sums of rearrangements corresponding to *double cosets* ${\mathcal {D}}_n \alpha {\mathcal {D}}_n$, $\alpha \in {{\,\mathrm{{\mathcal {H}}}\,}}_n$. An element of one of these double cosets looks like $d_1\alpha d_2$, with $d_1,d_2 \in {\mathcal {D}}_n$. Now, we can choose $\alpha d(d_2)^{-1}$ in the double coset, such that $d_2 \sigma$ is mapped to the same genome as $d\sigma$. Using formal sums instead of double cosets allows one to keep track of the probabilities of the possible outcomes when a rearrangement is applied to a genome. To allow ourselves to take sums of group elements, we move from the group itself to the group algebra ${\mathbb {C}}[{{\,\mathrm{{\mathcal {H}}}\,}}_n]$—a vector space (over the complex numbers, although in practice our coefficients will always lie between 0 and 1) which has the group as its basis, where we also allow ourselves to take products of elements. The product is simply the same as in the group, and is distributed over addition. As an example, we will describe the genome represented by $\sigma = [\hspace{0.55542pt}\overline{\hspace{-0.55542pt}1\hspace{-0.55542pt}}\hspace{0.55542pt}\ 4\ 3\ \hspace{0.55542pt}\overline{\hspace{-0.55542pt}5\hspace{-0.55542pt}}\hspace{0.55542pt}\ \hspace{0.55542pt}\overline{\hspace{-0.55542pt}2\hspace{-0.55542pt}}\hspace{0.55542pt}]$, used as an example in Fig. 2 as an element of the group algebra.

Define the *symmetry element*,

$$\begin{aligned} {\textbf{z}}:= \tfrac{1}{2n} \sum _{d \in {\mathcal {D}}_n} d \ \in \ {\mathbb {C}}[{{\,\mathrm{{\mathcal {H}}}\,}}_n] \end{aligned}$$

where the factor of $\frac{1}{2n}$ is so that the coefficients of the permutations in the sum all add to 1. For example, take the instance $\sigma = [\hspace{0.55542pt}\overline{\hspace{-0.55542pt}1\hspace{-0.55542pt}}\hspace{0.55542pt}\ 4\ 3\ \hspace{0.55542pt}\overline{\hspace{-0.55542pt}5\hspace{-0.55542pt}}\hspace{0.55542pt}\ \hspace{0.55542pt}\overline{\hspace{-0.55542pt}2\hspace{-0.55542pt}}\hspace{0.55542pt}]$. The product,

$$\begin{aligned} {\textbf{z}}\sigma = \sum _{d \in {\mathcal {D}}_n} d\sigma = \frac{1}{10}&\Big ([1\ \hspace{0.55542pt}\overline{\hspace{-0.55542pt}3\hspace{-0.55542pt}}\hspace{0.55542pt}\ \hspace{0.55542pt}\overline{\hspace{-0.55542pt}4\hspace{-0.55542pt}}\hspace{0.55542pt}\ 2\ 5] + [2\ \hspace{0.55542pt}\overline{\hspace{-0.55542pt}4\hspace{-0.55542pt}}\hspace{0.55542pt}\ \hspace{0.55542pt}\overline{\hspace{-0.55542pt}5\hspace{-0.55542pt}}\hspace{0.55542pt}\ 3\ 1]+ [3\ \hspace{0.55542pt}\overline{\hspace{-0.55542pt}5\hspace{-0.55542pt}}\hspace{0.55542pt}\ \hspace{0.55542pt}\overline{\hspace{-0.55542pt}1\hspace{-0.55542pt}}\hspace{0.55542pt}\ 4\ 2]+ [4\ \hspace{0.55542pt}\overline{\hspace{-0.55542pt}1\hspace{-0.55542pt}}\hspace{0.55542pt}\ \hspace{0.55542pt}\overline{\hspace{-0.55542pt}2\hspace{-0.55542pt}}\hspace{0.55542pt}\ 5\ 3]\\&\quad +\ [5\ \hspace{0.55542pt}\overline{\hspace{-0.55542pt}2\hspace{-0.55542pt}}\hspace{0.55542pt}\ \hspace{0.55542pt}\overline{\hspace{-0.55542pt}3\hspace{-0.55542pt}}\hspace{0.55542pt}\ 1\ 4]+ [\hspace{0.55542pt}\overline{\hspace{-0.55542pt}1\hspace{-0.55542pt}}\hspace{0.55542pt}\ 4\ 3\ \hspace{0.55542pt}\overline{\hspace{-0.55542pt}5\hspace{-0.55542pt}}\hspace{0.55542pt}\ \hspace{0.55542pt}\overline{\hspace{-0.55542pt}2\hspace{-0.55542pt}}\hspace{0.55542pt}]+ [\hspace{0.55542pt}\overline{\hspace{-0.55542pt}2\hspace{-0.55542pt}}\hspace{0.55542pt}\ 5\ 4\ \hspace{0.55542pt}\overline{\hspace{-0.55542pt}1\hspace{-0.55542pt}}\hspace{0.55542pt}\ \hspace{0.55542pt}\overline{\hspace{-0.55542pt}3\hspace{-0.55542pt}}\hspace{0.55542pt}]+ [\hspace{0.55542pt}\overline{\hspace{-0.55542pt}3\hspace{-0.55542pt}}\hspace{0.55542pt}\ 1\ 5\ \hspace{0.55542pt}\overline{\hspace{-0.55542pt}2\hspace{-0.55542pt}}\hspace{0.55542pt}\ \hspace{0.55542pt}\overline{\hspace{-0.55542pt}4\hspace{-0.55542pt}}\hspace{0.55542pt}]\\&\quad +\ [\hspace{0.55542pt}\overline{\hspace{-0.55542pt}4\hspace{-0.55542pt}}\hspace{0.55542pt}\ 2\ 1\ \hspace{0.55542pt}\overline{\hspace{-0.55542pt}3\hspace{-0.55542pt}}\hspace{0.55542pt}\ \hspace{0.55542pt}\overline{\hspace{-0.55542pt}5\hspace{-0.55542pt}}\hspace{0.55542pt}]+ [\hspace{0.55542pt}\overline{\hspace{-0.55542pt}5\hspace{-0.55542pt}}\hspace{0.55542pt}\ 3\ 2\ \hspace{0.55542pt}\overline{\hspace{-0.55542pt}4\hspace{-0.55542pt}}\hspace{0.55542pt}\ \hspace{0.55542pt}\overline{\hspace{-0.55542pt}1\hspace{-0.55542pt}}\hspace{0.55542pt}]\Big ), \end{aligned}$$

encodes every instance of the genome represented by $\sigma$.

The *genome algebra*, defined by Terauds and Sumner (2018), is the sub-algebra of ${\mathbb {C}}[{{\,\mathrm{{\mathcal {H}}}\,}}_n]$ spanned by the set of genomes ${\textbf{z}}\!{{\,\mathrm{{\mathcal {H}}}\,}}_n:= \{{\textbf{z}}\sigma : \sigma \in {{\,\mathrm{{\mathcal {H}}}\,}}_n\}$ (we can think of the basis as corresponding to the set of cosets ${{\,\mathrm{{\mathcal {H}}}\,}}_n/{\mathcal {D}}_n$). Similarly, the analogue of the double coset ${\mathcal {D}}_n \alpha {\mathcal {D}}_n$ is the algebra element ${\textbf{z}}\alpha {\textbf{z}}$. Formally, a rearrangement ${\textbf{z}}\alpha$ acts on a genome ${\textbf{z}}\sigma$ via multiplication, producing a weighted formal sum $({\textbf{z}}\alpha ) ({\textbf{z}}\sigma ) = ({\textbf{z}}\alpha {\textbf{z}})\sigma$ of genomes, which represents the possible outcomes of the rearrangement event, along with the probability of each outcome occurring. It is also helpful to view this expression as,

$$\begin{aligned} \begin{aligned} ({{\textbf {z}}}\alpha ) ({{\textbf {z}}}\sigma ){}&{} = {{\textbf {z}}}(\alpha ({{\textbf {z}}}\sigma ))\\&= \text{ all } \text{ orientations } \text{ of } \\&\qquad (\alpha \, \text {acting on (all orientations of the instance } \sigma )). \end{aligned} \end{aligned}$$

Two rearrangements ${\textbf{z}}\alpha$ and ${\textbf{z}}\beta$ applied to the genome ${\textbf{z}}$ will produce the same distribution of outcomes (that is, they apply the same action) precisely when ${\textbf{z}}\alpha {\textbf{z}} = {\textbf{z}}\beta {\textbf{z}}$, or equivalently when $\beta \in {\mathcal {D}}_n\alpha {\mathcal {D}}_n$. This set is the set of permutations generated by left multiplication by ${\mathcal {D}}_n$ (representing flips and rotations of the resulting genome) and conjugation by ${\mathcal {D}}_n$ (allowing the rearrangement to occur at any position on the genome, since we have no privileged positions). We refer to a set of allowed rearrangement actions ${\mathcal {M}} = \{ {\textbf{z}}\alpha _1 {\textbf{z}},\ldots , {\textbf{z}}\alpha _m {\textbf{z}} \}$ along with a probability map $w: {\mathcal {M}} \rightarrow [0,1]$ as a *model* of genome rearrangement.

#### Remark 2.1

We note that in a number of situations, for example our combinatorial analysis of rearrangements in Sect. 4, it is sufficient to simply think of rearrangements as double cosets, or more simply, sets of permutations which represent a given rearrangement, along with some number of flips or rotations applied before or after the rearrangement itself. While imagining genomes and rearrangements as formal sums belonging to some algebra (such as the genome algebra) allows us to easily keep track of model probabilities and the outcomes of rearrangement actions, as we will see in Sect. 3, fundamentally we are simply collecting all instances of the genome or rearrangement which we consider to be equivalent up to symmetry.

## Rearrangement models

**Table 2:** Some examples of classes of allowed rearrangements appearing in the literature

Model	References	Comment
*All inversions*	Hannenhalli and Pevzner (1996), Caprara (1997), Berard et al. (2007), Bafna and Pevzner (1996), Bader et al. (2001), Baudet et al. (2015), Lin and Moret (2008)	Inversions take a segment of regions and invert it in place, reversing the order of the regions in the segment, and switching their orientations. These rearrangements are also referred to as *reversals* and are used to compute ‘reversal distance’ or ‘inversion distance’. Inversions are discussed further in Sect. 3.1.
*All transpositions*	Bafna and Pevzner (1998), Alexandrino et al. (2020), Walter et al. (2005)	Transpositions move a segment of regions to another location in the genome. Some authors allow this section to be inverted in the same step (inverted transposition). It is worth noting that if inverted transpositions are not included in the unsigned case, not every genome will be reachable from the reference. Transpositions are discussed further in Sect. 3.2.
*Block-interchanges*	Huang et al. (2010)	Block-interchanges swap two non-intersecting segments of some number of regions, with or without inverting one or both of the segments.
*Inversions and Transpositions*	Lin and Xue (2001)	While transposition distance is less well-studied than inversion distance, some authors consider inversions and transpositions together.
*Inversions of one or two regions*	Oliveira et al. (2019), Galvao et al. (2017)	Due to biological evidence that the probability of an inversion occurring can depend on the size of the segment being inverted (Darling et al. 2008), some authors consider models consisting of these small rearrangements. Inversions of one or two regions are referred to as ‘Super-short reversals’.
*Transpositions of one or two regions*	Oliveira et al. (2019)	A similar model to above, but the operation is a transposition rather than an inversion. These are referred to as ‘Super-short transpositions’.
*Inversions or transpositions of one or two regions*	Oliveira et al. (2018, 2019)	Inversions and transpositions where the section of regions being operated on consists of either one or two regions. These rearrangements are referred to as ‘Super-short’ operations.
*Inversions of up to three regions*	Terauds and Sumner (2018)	Super-short reversals, except sections of three regions can also be inverted. Since only unsigned genomes were discussed by Terauds and Sumner (2018), the single-region inversions leave the genome unchanged.

It is widely accepted by biologists that some genomic rearrangement events are more likely than others (Dalevi et al. 2002; Lefebvre et al. 2003; Darling et al. 2008; Alexeev et al. 2015) [although there has been much debate over where the most likely locations are for rearrangements to occur (Peng et al. 2006; Pevzner and Tesler 2003)]. Some rearrangement event modelling approaches allow for only a specific subset of rearrangements, while others are valid for any subset. In this section we will review types of rearrangements commonly seen in the literature. A summary of commonly used models is given in Table 2.

### Inversions

*Inversions* are rearrangements that take a segment of regions and invert it in place, reversing the order of the regions in the segment, and switching their orientations. One of the most commonly seen sets of rearrangements considered in the literature is the set of all inversions (Bader et al. 2001; Watterson et al. 1982; Hannenhalli and Pevzner 1996). The minimum number of rearrangements needed to transform one genome into another under this model is usually referred to as the ‘inversion distance’ or ‘reversal distance’. In the position paradigm, we can give explicit algebraic characterisations for inversions, as follows.

#### Proposition 3.1

Fixing a position labelling for a genome with *n* oriented regions, inversions of odd length $2k+1$ about a signed region in position *r* can be written as the product of 2-cycles

$$\begin{aligned} \textrm{Inv}_n(r, 2k+1):= (r, \hspace{0.55542pt}\overline{\hspace{-0.55542pt}r\hspace{-0.55542pt}}\hspace{0.55542pt})\prod _{i=1}^k \big (r\!-\!i, \hspace{0.55542pt}\overline{\hspace{-0.55542pt}r\!+\!i\hspace{-0.55542pt}}\hspace{0.55542pt}\big ) \big (\hspace{0.55542pt}\overline{\hspace{-0.55542pt}r\!-\!i\hspace{-0.55542pt}}\hspace{0.55542pt}, r\!+\!i\big ) \in {{\,\mathrm{{\mathcal {H}}}\,}}_n, \end{aligned}$$

where all addition is $(\textrm{mod}\ n)$, and any zeros are replaced with *n* in the final expression, since we index positions from one, rather than from zero. Inversions of even length 2*k*, about a pair of regions in positions *r* and $r\ (\textrm{mod}\ n) + 1$ can be written

$$\begin{aligned} \textrm{Inv}_n(r, 2k):= \prod _{i=1}^k \big (r\!-\!(i\!-\!1), \hspace{0.55542pt}\overline{\hspace{-0.55542pt}r\!+\!i\hspace{-0.55542pt}}\hspace{0.55542pt}\big )\big (\hspace{0.55542pt}\overline{\hspace{-0.55542pt}r\!-\!(i\!-\!1)\hspace{-0.55542pt}}\hspace{0.55542pt}, r\!+\!i\big ) \in {{\,\mathrm{{\mathcal {H}}}\,}}_n, \end{aligned}$$

where again, all addition is $(\textrm{mod}\ n)$ and zeros are replaced with *n* in the final expression. $\square$

The set of all two region inversions generates the symmetric group, and the set of all one or two region inversions generates ${{\,\mathrm{{\mathcal {H}}}\,}}_n$.

### Transpositions

Compared to inversions, transpositions are less consistently defined in the literature. In this paper, a *transposition* is a rearrangement that takes a segment of regions and moves it somewhere else in the genome, with or without also inverting it. Some authors explicitly distinguish between the case where the segment is inverted and the case where it is not, referring to the prior as a *transreversal* (a transposition with reversal) (Alexandrino et al. 2020) or simply as an *inverted transposition*. Another way of defining a transposition is as a rearrangement that swaps two adjacent segments of regions. In this case, the transposition is thought of as ‘acting on’ both segments, and so either segment can be inverted. It’s worth noting that this definition is equivalent. We do not consider inverting *both* of the swapped segments as a transposition, as this rearrangement is equivalent to an inversion, and is indeed often ignored in the literature when considering transposition distance (Lin and Xue 2001; Alexandrino et al. 2020). Lin and Xue (2001) also consider the rearrangement that inverts two segments simultaneously *without* swapping them, considering this to be a third type of transposition (along with the standard transposition, and the inverted transposition). These are referred to as *revrevs* by Alexandrino et al. (2020). We can safely ignore this type of rearrangement, since in the case of circular genomes with dihedral symmetry, they are equivalent to transpositions with inversion.

Transpositions can be written as sequences of inversions. For example, to move a single region from position *r* to position $r+1$, simply invert the segment of both regions, and then invert each of the regions separately. In the notation provided in Proposition 3.1, this can be written as,

$$\begin{aligned} \textrm{Inv}_n(r+1, 1)\textrm{Inv}_n(r, 1)\textrm{Inv}_n(r, 2). \end{aligned}$$

Once transpositions are added to the model as independent rearrangements, however, there is more to think about in terms of their assigned probability and further rearrangements which may need to be added as a result. For example, does it make sense to include a transposition, but not include the same transposition that also inverts the segment(s) involved? In Sect. 3.3 we discuss questions such as this one and provide some examples. In each example, we describe a set of possible rearrangements, along with a relative probability. This information can be combined to form a Markov chain in which the states are the genomes themselves, or used with some other method for computing rearrangement distance.

### Examples

Identifying biologically realistic models for genome rearrangements along with associated probabilities is still a work in progress, and a model that is realistic for one group of organisms may not be for another. It is therefore important for any proposed framework to allow for a range of different models. The following examples will show some commonly studied models (for example ‘all inversions’) in the genome algebra framework. We will discuss some potential pitfalls and important considerations which arise when defining a model and choosing rearrangement probabilities.

#### Example 3.2

**Fig. 3:**
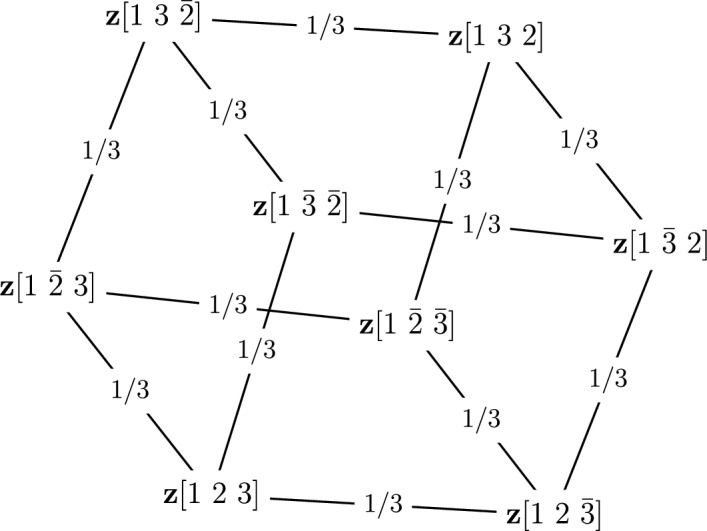
A graph representation of the Markov chain for a model of inversions on circular genomes with 3 oriented regions. Node labels are of the form ${\textbf{z}}\sigma$ where ${\textbf{z}}$ is the symmetry element and $\sigma$ is one particular instance of the genome (position paradigm, region $\rightarrow$ position) written in one-row notation. Lines between genomes show the probability of a rearrangement which transforms one into the other. The minimum number of steps needed to traverse the graph from one genome to another is the ‘inversion distance’. The edge weights are the same for any choice of probability mapping *w*, since with only three regions, every inversion of two regions can be written as an inversion of a single region

(All inversions, all equally likely) Suppose we wish to construct a model equivalent to that used to compute ‘inversion distance’ on circular genomes; that is, a model containing every inversion, with each inversion having an equal probability of occurring. See Fig. 3 for a visual representation of such a model.

Begin by including the inversion represented by $(1\hspace{0.55542pt}\overline{\hspace{-0.55542pt}1\hspace{-0.55542pt}}\hspace{0.55542pt})$ (an inversion of the single region in position 1). The action of the rearrangement is the same as the action of $(2\hspace{0.55542pt}\overline{\hspace{-0.55542pt}2\hspace{-0.55542pt}}\hspace{0.55542pt})$, for example, since the genome can be rotated or flipped before and after applying the rearrangement due to dihedral symmetry. As previously discussed, the sum of permutations ${\textbf{z}}(1\hspace{0.55542pt}\overline{\hspace{-0.55542pt}1\hspace{-0.55542pt}}\hspace{0.55542pt}) {\textbf{z}}$ represents the single region inversion action, and thus accounts for all such inversions. Similarly, ${\textbf{z}}(1\hspace{0.55542pt}\overline{\hspace{-0.55542pt}2\hspace{-0.55542pt}}\hspace{0.55542pt})(\hspace{0.55542pt}\overline{\hspace{-0.55542pt}1\hspace{-0.55542pt}}\hspace{0.55542pt}2){\textbf{z}}$ accounts for every inversion of 2 adjacent regions, and ${\textbf{z}}(1\hspace{0.55542pt}\overline{\hspace{-0.55542pt}3\hspace{-0.55542pt}}\hspace{0.55542pt})(2\hspace{0.55542pt}\overline{\hspace{-0.55542pt}2\hspace{-0.55542pt}}\hspace{0.55542pt})(\hspace{0.55542pt}\overline{\hspace{-0.55542pt}1\hspace{-0.55542pt}}\hspace{0.55542pt}3){\textbf{z}}$ gives us all of the 3-region inversions. Care must be taken for larger inversions, however, since for a circular genome with *n* regions, the $(n\!-\!m)$-region inversion action is the same as the *m*-region inversion action. If $n=6$, for example, the three inversions we have mentioned so far account for all inversions. If $n=5$, for equal probabilities we must remove the 3 region inversion ${\textbf{z}}(1\hspace{0.55542pt}\overline{\hspace{-0.55542pt}3\hspace{-0.55542pt}}\hspace{0.55542pt})(2\hspace{0.55542pt}\overline{\hspace{-0.55542pt}2\hspace{-0.55542pt}}\hspace{0.55542pt})(\hspace{0.55542pt}\overline{\hspace{-0.55542pt}1\hspace{-0.55542pt}}\hspace{0.55542pt}3){\textbf{z}}$ as it is equivalent to an inversion of two regions and would therefore skew the probability distribution of outcomes if included. The effect that this can have on subsequent calculations of genomic distance is illustrated in Terauds et al. (2021).

It is important to note that for $n\!=\!6$, the model

$$\begin{aligned} {\mathcal {M}} = \{ {\textbf{z}}(1\hspace{0.55542pt}\overline{\hspace{-0.55542pt}1\hspace{-0.55542pt}}\hspace{0.55542pt}) {\textbf{z}}, {\textbf{z}}(1\hspace{0.55542pt}\overline{\hspace{-0.55542pt}2\hspace{-0.55542pt}}\hspace{0.55542pt})(\hspace{0.55542pt}\overline{\hspace{-0.55542pt}1\hspace{-0.55542pt}}\hspace{0.55542pt}2){\textbf{z}}, {\textbf{z}}(1\hspace{0.55542pt}\overline{\hspace{-0.55542pt}3\hspace{-0.55542pt}}\hspace{0.55542pt})(2\hspace{0.55542pt}\overline{\hspace{-0.55542pt}2\hspace{-0.55542pt}}\hspace{0.55542pt})(\hspace{0.55542pt}\overline{\hspace{-0.55542pt}1\hspace{-0.55542pt}}\hspace{0.55542pt}3){\textbf{z}} \} \end{aligned}$$

with an equal weighting of $\frac{1}{3}$ for each action will produce (via the process detailed by Terauds and Sumner (2018)) a Markov chain in which some outcomes reachable via a single step will be more likely than others. This is because the action of inverting 3 regions has 3 possible outcomes, whereas each of the other inversion actions have 6. In other words, the sum ${\textbf{z}}(1\hspace{0.55542pt}\overline{\hspace{-0.55542pt}3\hspace{-0.55542pt}}\hspace{0.55542pt})(2\hspace{0.55542pt}\overline{\hspace{-0.55542pt}2\hspace{-0.55542pt}}\hspace{0.55542pt})(\hspace{0.55542pt}\overline{\hspace{-0.55542pt}1\hspace{-0.55542pt}}\hspace{0.55542pt}3){\textbf{z}}$ has fewer terms. The model for which *all reachable genomes are equally likely* can be achieved via assigning the probability $\frac{2}{5}$ to the one and two region inversions, and $\frac{1}{5}$ to the three region inversions.

Note that in total, there are $\left\lfloor \frac{n}{2}\right\rfloor$ distinct inversion actions for a genome with *n* regions. This is shown by Proposition 4.8.

#### Example 3.3

(One and two region inversions) A model containing small inversions of one or two regions can be defined with the two sums of permutations, ${\textbf{z}}(1\hspace{0.55542pt}\overline{\hspace{-0.55542pt}1\hspace{-0.55542pt}}\hspace{0.55542pt}) {\textbf{z}}$ and ${\textbf{z}}(1\hspace{0.55542pt}\overline{\hspace{-0.55542pt}2\hspace{-0.55542pt}}\hspace{0.55542pt})(\hspace{0.55542pt}\overline{\hspace{-0.55542pt}1\hspace{-0.55542pt}}\hspace{0.55542pt}2){\textbf{z}}$, as above. Since the number of regions *n* is encoded in the symmetry element ${\textbf{z}}$, this model can be written in the same way for any number of regions $n > 2$. These rearrangement actions can be allowed to occur with equal probability by simply assigning $\frac{1}{2}$ to each action, or they can be given different weights. For example, there is some evidence of smaller inversions being more prevalent compared to larger inversions (Dalevi et al. 2002; Lefebvre et al. 2003), and this can be modelled by assigning the 1- and 2-region inversions the probabilities $\frac{2}{3}$ and $\frac{1}{3}$, for example. In this case, the 1-region inversion is twice as likely to occur.

#### Example 3.4

(One and two region inversions, and two region adjacent transpositions) Suppose we have a model with small (1- and 2-region) inversions as in the previous examples. We can add adjacent transpositions (rearrangements that swap two regions) by including the additional element ${\textbf{z}}(12)(\hspace{0.55542pt}\overline{\hspace{-0.55542pt}1\hspace{-0.55542pt}}\hspace{0.55542pt}\hspace{0.55542pt}\overline{\hspace{-0.55542pt}2\hspace{-0.55542pt}}\hspace{0.55542pt}) {\textbf{z}}$. In this situation, it might make sense to also allow inverting one or both of these swapped regions in a single step. The action ${\textbf{z}}(12\hspace{0.55542pt}\overline{\hspace{-0.55542pt}1\hspace{-0.55542pt}}\hspace{0.55542pt}\hspace{0.55542pt}\overline{\hspace{-0.55542pt}2\hspace{-0.55542pt}}\hspace{0.55542pt}) {\textbf{z}}$ swaps regions in adjacent positions, while simultaneously inverting the second region (the action that inverts the first region is equivalent due to symmetry). In this case, one should consider a weighting for this new action that makes sense considering the other weightings.

For example, if $w({\textbf{z}}(1\hspace{0.55542pt}\overline{\hspace{-0.55542pt}1\hspace{-0.55542pt}}\hspace{0.55542pt}) {\textbf{z}}) = a$ and $w({\textbf{z}}(12)(\hspace{0.55542pt}\overline{\hspace{-0.55542pt}1\hspace{-0.55542pt}}\hspace{0.55542pt}\hspace{0.55542pt}\overline{\hspace{-0.55542pt}2\hspace{-0.55542pt}}\hspace{0.55542pt}) {\textbf{z}}) = b$, then it might make sense to require that $w({\textbf{z}}(12\hspace{0.55542pt}\overline{\hspace{-0.55542pt}1\hspace{-0.55542pt}}\hspace{0.55542pt}\hspace{0.55542pt}\overline{\hspace{-0.55542pt}2\hspace{-0.55542pt}}\hspace{0.55542pt}) {\textbf{z}}) \ge ab$. One can think of this property as the triangle inequality for a rearrangement model.

#### Example 3.5

Another commonly considered class of rearrangement events is the collection of transpositions. These are rearrangements which cut a segment from the genome and move it to a new location and/or orientation. In the previous example, ${\textbf{z}}(12\hspace{0.55542pt}\overline{\hspace{-0.55542pt}1\hspace{-0.55542pt}}\hspace{0.55542pt}\hspace{0.55542pt}\overline{\hspace{-0.55542pt}2\hspace{-0.55542pt}}\hspace{0.55542pt}) {\textbf{z}}$ and ${\textbf{z}}(12)(\hspace{0.55542pt}\overline{\hspace{-0.55542pt}1\hspace{-0.55542pt}}\hspace{0.55542pt}\hspace{0.55542pt}\overline{\hspace{-0.55542pt}2\hspace{-0.55542pt}}\hspace{0.55542pt}){\textbf{z}}$ were examples of transpositions of adjacent regions. Another permutation representing a transposition is $(132)(\hspace{0.55542pt}\overline{\hspace{-0.55542pt}1\hspace{-0.55542pt}}\hspace{0.55542pt}\hspace{0.55542pt}\overline{\hspace{-0.55542pt}3\hspace{-0.55542pt}}\hspace{0.55542pt}\hspace{0.55542pt}\overline{\hspace{-0.55542pt}2\hspace{-0.55542pt}}\hspace{0.55542pt})$, which takes the region in position 1 and moves it to position 3. More generally, the rearrangement action ${\textbf{z}}(132)(\hspace{0.55542pt}\overline{\hspace{-0.55542pt}1\hspace{-0.55542pt}}\hspace{0.55542pt}\hspace{0.55542pt}\overline{\hspace{-0.55542pt}3\hspace{-0.55542pt}}\hspace{0.55542pt}\hspace{0.55542pt}\overline{\hspace{-0.55542pt}2\hspace{-0.55542pt}}\hspace{0.55542pt}){\textbf{z}}$ takes a region and moves it to the location two regions further along the genome. There are many more transposition actions. For $n=5$, we have 6 such actions: two correspond to moving a single region 1 or 2 places along the genome, and for each of these, we can invert the region being moved, or invert one of the other segments, or both the single region and one of the segments. Of course, when there are only 5 regions, moving a segment of two regions along the genome is equivalent to moving a single region. If inversions are included in the model as single-step rearrangements, one might want to remove transpositions which invert one of the segments. This can be done by simply removing rearrangements with cycles that contain positive and negative region labels (for example ${\textbf{z}}(12\hspace{0.55542pt}\overline{\hspace{-0.55542pt}1\hspace{-0.55542pt}}\hspace{0.55542pt}\hspace{0.55542pt}\overline{\hspace{-0.55542pt}2\hspace{-0.55542pt}}\hspace{0.55542pt}){\textbf{z}}$). We will examine these rearrangements more closely in Sect. 4, where we count the distinct rearrangements of this type.

## Cuts and breakpoints

**Fig. 4:**
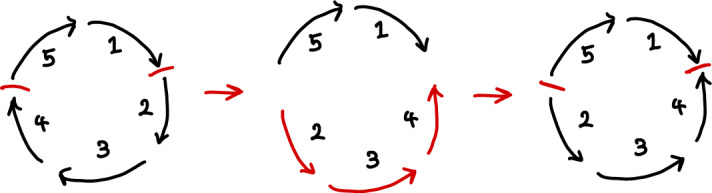
An inversion represented by the permutation $\alpha = (3\hspace{0.55542pt}\overline{\hspace{-0.55542pt}3\hspace{-0.55542pt}}\hspace{0.55542pt})(2\hspace{0.55542pt}\overline{\hspace{-0.55542pt}4\hspace{-0.55542pt}}\hspace{0.55542pt})(\hspace{0.55542pt}\overline{\hspace{-0.55542pt}2\hspace{-0.55542pt}}\hspace{0.55542pt}4)$ (as in Example 4.3), applied to the reference genome (Color figure online)

While the examples in the previous section provide some intuition for defining genome rearrangement models, it can be useful to formally describe possible rearrangements in order to develop a clearer picture. The rearrangements discussed so far have all been defined by describing a process wherein one or more sections of the genome are cut out and then added back in. In this section, we consider the categorisation of rearrangements via the positions of the required cuts in the genome, and find expressions for the numbers of distinct rearrangements that require two and three cuts. For example, given a position labelling, the permutation $\alpha = (3\hspace{0.55542pt}\overline{\hspace{-0.55542pt}3\hspace{-0.55542pt}}\hspace{0.55542pt})(2\hspace{0.55542pt}\overline{\hspace{-0.55542pt}4\hspace{-0.55542pt}}\hspace{0.55542pt})(\hspace{0.55542pt}\overline{\hspace{-0.55542pt}2\hspace{-0.55542pt}}\hspace{0.55542pt}4)$ represents an instance of an inversion which cuts the genome in two places. That is, between positions 1 and 2 and positions 4 and 5, as shown in Fig. 4. Formally, consider the following definition of the set of required cut positions for a rearrangement.

### Definition 4.1

For $\alpha \in {{\,\mathrm{{\mathcal {H}}}\,}}_n$, the *set of cuts required by* $\alpha$ is given by,

$$\begin{aligned} {{\,\mathrm{\textrm{CutPos}}\,}}(\alpha ):= \left\{ \text {positions } i\in [n]\ \Bigg \vert \begin{array}{l} \text {the application of } \alpha \,\, \text {requires cutting a }\\ \text {genome between positions } \,\, i \,\, \text {and } \,\, i \,\, \ (\text {mod}\ n) + 1 \end{array} \right\} . \end{aligned}$$

This idea is similar to the important (although somewhat overloaded, with widely differing definitions) term, *breakpoint*. In short, the breakpoints are the regions in the genome at which rearrangements can or have occurred. We will discuss breakpoints and the similarities to Definition 4.1 later in this section.

Recall the permutation $c=(1,\ldots ,n)(\hspace{0.55542pt}\overline{\hspace{-0.55542pt}n\hspace{-0.55542pt}}\hspace{0.55542pt},\ldots ,\hspace{0.55542pt}\overline{\hspace{-0.55542pt}1\hspace{-0.55542pt}}\hspace{0.55542pt}) \in {\mathcal {S}}_{[\hspace{0.55542pt}\overline{\hspace{-0.55542pt}n\hspace{-0.55542pt}}\hspace{0.55542pt}]}$, along with the following definitions. The *commutator* of $\alpha$ and *c* is defined as $[\alpha ,c]:= \alpha ^{-1} c^{-1}\alpha c \in {{\,\mathrm{{\mathcal {H}}}\,}}_n$, and its *fix* is defined as $\textrm{Fix}([\alpha ,c])\!=\!\{ i\!\in \![n]: [\alpha ,c](i)\!=\!i \}$. We can now prove the following result.

### Proposition 4.2

For $\alpha \in {{\,\mathrm{{\mathcal {H}}}\,}}_n$, the *set of cuts* required by a rearrangement instance $\alpha$ is given by,

2 $$\begin{aligned} {{\,\mathrm{\textrm{CutPos}}\,}}(\alpha ):= [n] - \textrm{Fix}([\alpha ,c]). \end{aligned}$$

### Proof

Recall that, given any position *i*, the next position on a circular genome is *c*(*i*). That is, we can write the position $i\ (\textrm{mod}\ n) + 1$ simply as *c*(*i*).

Consider $\alpha \in {{\,\mathrm{{\mathcal {H}}}\,}}_n$. The rearrangement represented by $\alpha$ requires a cut in position *i* if and only if it maps the position *c*(*i*) to something other than $c(\alpha (i))$. This is because if $c(\alpha (i)) = \alpha (c(i))$, then the regions in the adjacent positions *i* and *c*(*i*) will stay together once the rearrangement is applied. Formally, we can write,

$$\begin{aligned} {{\,\mathrm{\textrm{CutPos}}\,}}(\alpha )&= \{ i \in [n] : c\alpha (i) \ne \alpha c (i) \} \\&= \{ i \in [n] : i \ne \alpha ^{-1}c^{-1}\alpha c(i) \} \\&= \left\{ i \in [n] : i\notin \textrm{Fix}([\alpha ,c])\right\} \\&= [n] - \textrm{Fix}([\alpha ,c]) \end{aligned}$$

as required. $$\square $$

### Example 4.3

As in Proposition 3.1, the permutation $\alpha = (3\hspace{0.55542pt}\overline{\hspace{-0.55542pt}3\hspace{-0.55542pt}}\hspace{0.55542pt})(2\hspace{0.55542pt}\overline{\hspace{-0.55542pt}4\hspace{-0.55542pt}}\hspace{0.55542pt})(\hspace{0.55542pt}\overline{\hspace{-0.55542pt}2\hspace{-0.55542pt}}\hspace{0.55542pt}4)$ represents a rearrangement that cuts a genome in two positions to invert the 3-region segment spanning positions 2, 3 and 4. We have

$$\begin{aligned}{}[\alpha , c]&= \alpha ^{-1}c^{-1}\alpha c \\&= (3\hspace{0.55542pt}\overline{\hspace{-0.55542pt}3\hspace{-0.55542pt}}\hspace{0.55542pt})(2\hspace{0.55542pt}\overline{\hspace{-0.55542pt}4\hspace{-0.55542pt}}\hspace{0.55542pt}) (\hspace{0.55542pt}\overline{\hspace{-0.55542pt}2\hspace{-0.55542pt}}\hspace{0.55542pt}4) (\hspace{0.55542pt}\overline{\hspace{-0.55542pt}1\hspace{-0.55542pt}}\hspace{0.55542pt}\hspace{0.55542pt}\overline{\hspace{-0.55542pt}2\hspace{-0.55542pt}}\hspace{0.55542pt}\hspace{0.55542pt}\overline{\hspace{-0.55542pt}3\hspace{-0.55542pt}}\hspace{0.55542pt}\hspace{0.55542pt}\overline{\hspace{-0.55542pt}4\hspace{-0.55542pt}}\hspace{0.55542pt}\hspace{0.55542pt}\overline{\hspace{-0.55542pt}5\hspace{-0.55542pt}}\hspace{0.55542pt})(54321)(3\hspace{0.55542pt}\overline{\hspace{-0.55542pt}3\hspace{-0.55542pt}}\hspace{0.55542pt})(2\hspace{0.55542pt}\overline{\hspace{-0.55542pt}4\hspace{-0.55542pt}}\hspace{0.55542pt}) (\hspace{0.55542pt}\overline{\hspace{-0.55542pt}2\hspace{-0.55542pt}}\hspace{0.55542pt}4) (12345)(\hspace{0.55542pt}\overline{\hspace{-0.55542pt}5\hspace{-0.55542pt}}\hspace{0.55542pt}\hspace{0.55542pt}\overline{\hspace{-0.55542pt}4\hspace{-0.55542pt}}\hspace{0.55542pt}\hspace{0.55542pt}\overline{\hspace{-0.55542pt}3\hspace{-0.55542pt}}\hspace{0.55542pt}\hspace{0.55542pt}\overline{\hspace{-0.55542pt}2\hspace{-0.55542pt}}\hspace{0.55542pt}\hspace{0.55542pt}\overline{\hspace{-0.55542pt}1\hspace{-0.55542pt}}\hspace{0.55542pt}) \\&= (1\hspace{0.55542pt}\overline{\hspace{-0.55542pt}5\hspace{-0.55542pt}}\hspace{0.55542pt})(4\hspace{0.55542pt}\overline{\hspace{-0.55542pt}2\hspace{-0.55542pt}}\hspace{0.55542pt}) \end{aligned}$$

which fixes $2, 3, 5, \hspace{0.55542pt}\overline{\hspace{-0.55542pt}1\hspace{-0.55542pt}}\hspace{0.55542pt}, \hspace{0.55542pt}\overline{\hspace{-0.55542pt}3\hspace{-0.55542pt}}\hspace{0.55542pt}$ and $\hspace{0.55542pt}\overline{\hspace{-0.55542pt}4\hspace{-0.55542pt}}\hspace{0.55542pt}$. Thus ${{\,\mathrm{\textrm{CutPos}}\,}}(\alpha ) = [n] - \textrm{Fix}([\alpha ,c]) = \{1,4\}$, as expected since $\alpha$ cuts the genome before position 1 and after position 4.

Note that Eq. (2) given in Proposition 4.2 is very similar to the expression for the number of breakpoints derived by Meidanis and Dias (2000). In fact, breakpoints are a similar concept, but are typically sets of regions rather than sets of positions. We will obtain an equivalent expression for the number of breakpoints in Sect. 4.3, using a similar construction to the cuts set.

As discussed in Sect. 2.2, in our formulation which incorporates circular symmetry, rearrangements aren’t defined to be single permutations, but rather cosets or formal sums. The following result shows that all instances of a given rearrangement produce the same cut set, and thus the cut set is well-defined on the set of rearrangements. We first need the following definition.

### Definition 4.4

For all permutations $\alpha \in {{\,\mathrm{{\mathcal {H}}}\,}}_n$, denote

$$\begin{aligned} {{\,\mathrm{\textrm{CutPos}*}\,}}(\alpha ):= {{\,\mathrm{\textrm{CutPos}}\,}}(\alpha ) \cup \hspace{0.55542pt}\overline{\hspace{-0.55542pt}{{\,\mathrm{\textrm{CutPos}}\,}}(\alpha )\hspace{-0.55542pt}}\hspace{0.55542pt}, \end{aligned}$$

where $$\ \hspace{0.55542pt}\overline{\hspace{-0.55542pt}{{\,\mathrm{\textrm{CutPos}}\,}}(\alpha )\hspace{-0.55542pt}}\hspace{0.55542pt}:= \{ \hspace{0.55542pt}\overline{\hspace{-0.55542pt}\,i\,\hspace{-0.55542pt}}\hspace{0.55542pt}: i \in {{\,\mathrm{\textrm{CutPos}}\,}}(\alpha ) \}$$.

Simply put, Definition 4.4 describes a set which includes *both* orientations of the positions in the cut set ${{\,\mathrm{\textrm{CutPos}}\,}}(\alpha )$. If we take $\alpha$ from Example 4.3, we get ${{\,\mathrm{\textrm{CutPos}*}\,}}(\alpha ) = \{1,4,\hspace{0.55542pt}\overline{\hspace{-0.55542pt}1\hspace{-0.55542pt}}\hspace{0.55542pt},\hspace{0.55542pt}\overline{\hspace{-0.55542pt}4\hspace{-0.55542pt}}\hspace{0.55542pt}\}$. This idea allows us to concisely state the following result.

### Proposition 4.5

For all permutations $\alpha \in {{\,\mathrm{{\mathcal {H}}}\,}}_n$ and $d_1,d_2\in {\mathcal {D}}_n$, we have,

$$\begin{aligned} {{\,\mathrm{\textrm{CutPos}*}\,}}(d_1\alpha d_2) = \{ d_2^{-1}(i): i \in {{\,\mathrm{\textrm{CutPos}*}\,}}(\alpha ) \} =: d_2^{-1}\cdot {{\,\mathrm{\textrm{CutPos}*}\,}}(\alpha ). \end{aligned}$$

Further,

$$\begin{aligned} {{\,\mathrm{\textrm{CutPos}}\,}}(d_1\alpha d_2) = ( {d_2^{-1}\cdot {{\,\mathrm{\textrm{CutPos}*}\,}}(\alpha )} ) \cap [n]. \end{aligned}$$

That is, if two rearrangements have the same action, then their cut sets are dihedrally related. In particular,

$$\begin{aligned} {{\,\mathrm{\textrm{CutPos}}\,}}(d_1\alpha ) = {{\,\mathrm{\textrm{CutPos}}\,}}(\alpha ). \end{aligned}$$

### Proof

Take some $\alpha \in {{\,\mathrm{{\mathcal {H}}}\,}}_n$ and $d_1,d_2\in {\mathcal {D}}_n$. Then,

$$\begin{aligned} \begin{aligned} {{\,\mathrm {\textrm{CutPos}*}\,}}(d_1\alpha d_2)&= \left\{ i\in [\hspace{0.55542pt}\overline{\hspace{-0.55542pt}n\hspace{-0.55542pt}}\hspace{0.55542pt}] : \ cd_1\alpha d_2(i) \ne d_1\alpha d_2c(i)\right\} \\&= \left\{ i\in [\hspace{0.55542pt}\overline{\hspace{-0.55542pt}n\hspace{-0.55542pt}}\hspace{0.55542pt}] : \ d_1^{-1}cd_1\alpha d_2(i) \ne \alpha d_2c(i)\right\} \\&= \left\{ i\in [\hspace{0.55542pt}\overline{\hspace{-0.55542pt}n\hspace{-0.55542pt}}\hspace{0.55542pt}] : \ c\alpha d_2(i) \ne \alpha c d_2(i)\right\} \qquad \qquad \quad ({\because c \text{ commutes } \text{ with } \text{ all } \text{ of } {\mathcal {D}}_n}) \\&= \left\{ d_2^{-1}(l) : l \in [\hspace{0.55542pt}\overline{\hspace{-0.55542pt}n\hspace{-0.55542pt}}\hspace{0.55542pt}] \text{ and } c\alpha (l) \ne \alpha c (l) \right\} \quad \quad ({\text{ writing } l := d_2(i)}\,)\\&= d_2^{-1}\cdot {{\,\mathrm {\textrm{CutPos}*}\,}}(\alpha ). \end{aligned} \end{aligned}$$

The second result is a simple application of the first, setting $d_2$ equal to the identity.

$$\square $$

Using the cuts set, we can now conveniently express sets of rearrangements in terms of the number of cuts required by each rearrangement.

### Lemma 4.6

The set of cut positions describes the following classes of rearrangements on circular, uni-chromosomal genomes with *n* oriented regions.

(i) Inversions of some number of adjacent regions:
  $$\begin{aligned} \textrm{SignedInversions}(n):= \{ {\textbf{z}}\alpha \in {\mathbb {C}}[{{\,\mathrm{{\mathcal {H}}}\,}}_n]: |{{\,\mathrm{\textrm{CutPos}}\,}}(\alpha )| = 2\} \end{aligned}$$
(ii) Transpositions, including where one of the segments is inverted:
  $$\begin{aligned} \textrm{SignedTranspositions}(n):= \{ {\textbf{z}}\alpha \in {\mathbb {C}}[{{\,\mathrm{{\mathcal {H}}}\,}}_n]: |{{\,\mathrm{\textrm{CutPos}}\,}}(\alpha )| = 3 \} \end{aligned}$$
(iii) Block interchanges (two segments are swapped and/or inverted):
  $$\begin{aligned} \textrm{SignedBlockInterchanges}(n):= \{ {\textbf{z}}\alpha \in {\mathbb {C}}[{{\,\mathrm{{\mathcal {H}}}\,}}_n]: |{{\,\mathrm{\textrm{CutPos}}\,}}(\alpha )| \in \{2,3,4\} \} \end{aligned}$$

### Proof

As an inversion needs to remove a section of regions and place it back into the same spot after reversing the order and orientation of the regions, two cuts are required for this kind of rearrangement. Conversely, if a rearrangement requires two cuts, the genome must have been broken in two and reformed. If either region is inverted in place, we have an inversion. If both regions invert, we simply have a reflection, which requires zero cuts. The only other option is that the regions swapped, and due to rotational symmetry, this is equivalent to an inversion of one of the segments.

Compared to an inversion, a transposition removes a section *and* places it in a different location, and so an additional cut is needed. Any rearrangement which requires three cuts means that the genome broke into three parts and then reformed. If only one of the segments inverts, only two cuts would have been required. If two regions swap places we have a transposition, unless both segments also invert at the same time, in which case we again have an inversion, which requires only two cuts. If all three segments are inverted, this is equivalent to a transposition.

Finally, a block-interchange swaps two segments (with or without inverting either or both of them). A block-interchange therefore requires three cuts if the two segments are next to each-other, two cuts if adjacent swapped segments are both inverted, or four cuts in all other cases. $\square$

### How many rearrangements ${\textbf{z}}\sigma$ require *k* cuts?

**Table 3:** Each entry *R*(*n*, *k*) in the above table is the number of distinct rearrangements ${\mathcal {D}}_n\alpha$ that require a given number of cuts

			# ${\mathcal {D}}_n\alpha$ requiring *k* cuts							
n	$|{{\,\mathrm{{\mathcal {H}}}\,}}_n|$	# ${\mathcal {D}}_n\alpha$	0	1	2	3	4	5	6	7
2	8	2	1	0	1					
3	48	8	1	0	3	4				
4	384	48	1	0	6	16	25			
5	3840	384	1	0	10	40	125	208		
6	46,080	3840	1	0	15	80	375	1248	2121	
7	645,120	46,080	1	0	21	140	875	4368	14,847	25,828

**Table 4:** Number of distinct rearrangements for each distinct cut-set of size *k*, calculated as $$\frac{ R(n,k)}{\left( {\begin{array}{c}n\\ k\end{array}}\right) }$$, where *R*(*n*, *k*) represents the number of rearrangements requiring *k* cuts for a genome with *n* regions, as given in Table 3

			# ${\mathcal {D}}_n\alpha$ per cut set of size *k*							
n	$|{{\,\mathrm{{\mathcal {H}}}\,}}_n|$	# ${\mathcal {D}}_n\alpha$	0	1	2	3	4	5	6	7
2	8	2	1	0	1					
3	48	8	1	0	1	4				
4	384	48	1	0	1	4	25			
5	3840	384	1	0	1	4	25	208		
6	46,080	3840	1	0	1	4	25	208	2121	
7	645,120	46,080	1	0	1	4	25	208	2121	25,828

The set of cut positions (which we will refer to as the cut-set for brevity) can be a useful way of thinking about rearrangements. We can first make the observation that the number of distinct cut-sets on *n* regions is simply the number of ways to break the circular genome into *k* segments, given by $$\left( {\begin{array}{c}n\\ k\end{array}}\right) $$. Let *R*(*n*, *k*) denote the number of rearrangements which require *k* cuts, which we have computed and displayed for a range of values *n* and *k* in Table 3. We wish to obtain an expression for *R*(*n*, *k*).

Since the number of ways to reconnect a circular genome cut into *k* segments is independent of the positions of the cuts and the number of regions *n*, we can simply write the number of different rearrangements for a given cut set as the total number of rearrangements requiring *k* cuts, divided by the number of possible cut-sets; that is, $$\frac{R(n,k)}{\left( {\begin{array}{c}n\\ k\end{array}}\right) }$$. The values of this expression for various *n* and *k* are presented in Table 4, where we can see that they are independent of *n* as expected, and for increasing *k* the sequence begins as:

$$\begin{aligned} 1,0,1,4,25,208,2121,25828,\ldots . \end{aligned}$$

We observe that this sequence appears to coincide with the sequence A061714 in the *Online Encyclopedia of Integer Sequences* (OEIS) (The O. E. I. S. Foundation 2020), and we establish in Proposition 4.7 that these sequences are indeed the same.

**Fig. 5:**
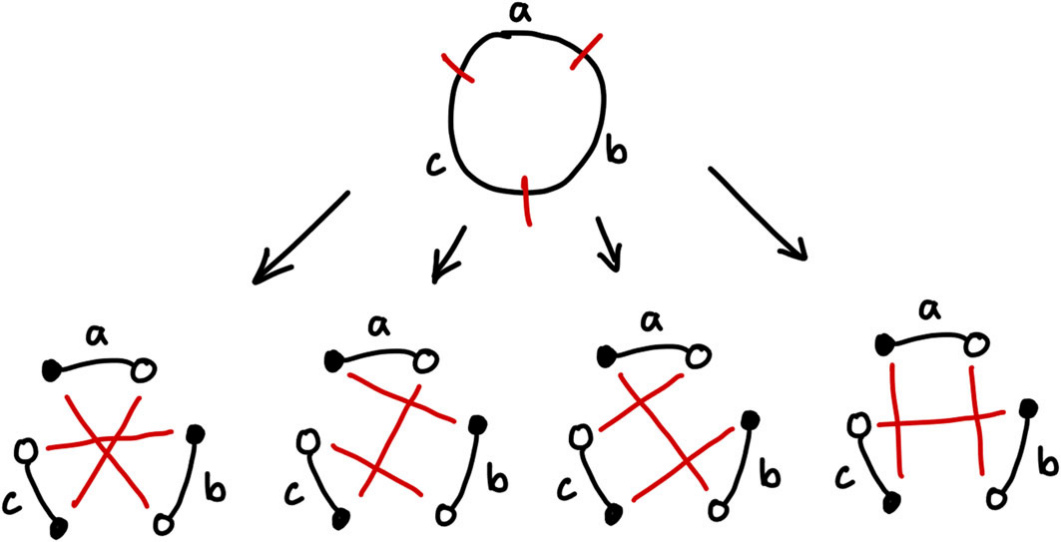
Different ways to reconnect three labelled segments to perform a rearrangement that requires 3 cuts (Color figure online)

We detour briefly to think about how we might count the number of rearrangements which correspond to a given cut set, for small *k*. Consider $k=3$, and consider some fixed set of cuts. Such a rearrangement will split a genome into three parts. The rearrangement will re-connect these segments in one of four different ways, as shown in Fig. 5.

**Fig. 6:**
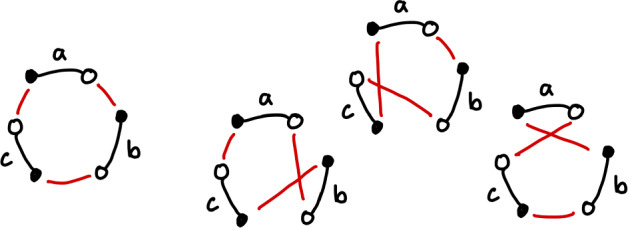
A diagram representing the identity rearrangement, and three representing rearrangements that require 2 cuts (Color figure online)

**Fig. 7:**
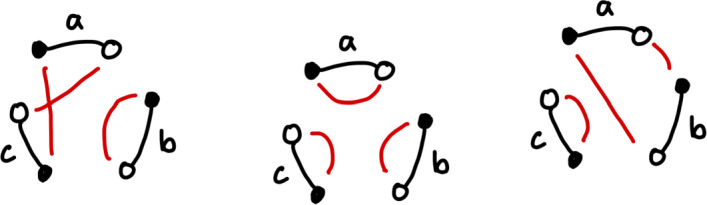
Diagrams representing fission rearrangements, which we do not consider here (Color figure online)

Of course, there are other ways to reconnect these segments, for example the graphs in Fig. 6. We do not wish to count these rearrangements. A rearrangement matching the first picture is simply the identity rearrangement, and the next three pictures represent rearrangements which require just two cuts, rather than three. We also do not count the rearrangements depicted in Fig. 7, as these represent fissions, which break a single chromosome genome into two, which we do not consider in this paper.

If one includes examples such as those shown in Figs. 5 and 6, it is easy to see that we obtain 8 possible rearrangements ($8=2^{3-1}(3-1)!$), which is the same as the total number of genomes with 3 regions. The OEIS sequence A061714, which has been connected to the travelling salesman problem by Helsgaun (2009), can be used to write the number of rearrangements which require precisely *k* cuts.

#### Proposition 4.7

The number of rearrangements ${\mathcal {D}}_n \alpha \in {{\,\mathrm{{\mathcal {H}}}\,}}_n/{\mathcal {D}}_n$ such that $|{{\,\mathrm{\textrm{CutPos}}\,}}(\alpha )| = k$ is

$$\begin{aligned} {R(n,k) = \left( {\begin{array}{c}n\\ k\end{array}}\right) }\textrm{A061714}(k) \ =\ {\left( {\begin{array}{c}n\\ k\end{array}}\right) } (-1)^{k}+ {\left( {\begin{array}{c}n\\ k\end{array}}\right) }\sum _{i=0}^{k-1}(-1)^{k-1+i}\left( \begin{array}{c}k \\ i+1\end{array}\right) i!\ 2^{i}. \end{aligned}$$

#### Proof

Let ${\mathcal {D}}_n \alpha$ be a rearrangement such that $|\!{{\,\mathrm{\textrm{CutPos}}\,}}(\alpha )| = k$. The rearrangement breaks a circular genome $\sigma$ into *k* segments. In the context of the travelling salesman problem, a tour is simply a collection of directed edges forming a path which visits each node exactly once. A *k*-opt move is a procedure which removes *k* edges from the tour and replaces them with *k* new edges which also form a tour, for example see Helsgaun (2009). In our context, we can consider the original genome as a tour, where the nodes are the *k* segments. The *k*-opt moves correspond to precisely the rearrangements shown in Figs. 5 and 6. Further, *pure* *k*-opt moves are *k*-opt moves for which the new set of edges and the old set of edges are disjoint. These correspond precisely to the rearrangements shown in Fig. 5. Helsgaun (2009) identifies the sequence $\textrm{A061714}$ with the number of *pure* *k*-*opt moves*, and so we have

$$\begin{aligned} \frac{R(n,k)}{\left( {\begin{array}{c}n\\ k\end{array}}\right) } = \textrm{A061714}(k) \implies R(n,k) = {\left( {\begin{array}{c}n\\ k\end{array}}\right) } \textrm{A061714}(k), \end{aligned}$$

as required. $$\square $$

The interested reader can verify via the generating function for $\textrm{A061714}$ that the sum of the above expression over all positive integer values of *k* is equal to the number of cosets ${{\,\mathrm{{\mathcal {H}}}\,}}_n/{\mathcal {D}}_n$.

### How many distinct *k*-cut rearrangement actions are there?

As described in Sect. 2.2, it makes sense to think about rearrangement actions as algebra elements corresponding to double cosets. Proposition 4.5 shows that each double coset admits a set of sets of cut positions which are all of the same size. We can therefore identify distinct rearrangement actions which require a given number of cuts. The number of actions requiring *k* cuts for *n* regions, $n \le 7$ is summarised in Table 5.

While we have no closed form expression for the number of such actions, we can now write the number of distinct rearrangement actions requiring 2 or 3 cuts. These respectively correspond to inversions and transpositions (Lemma 4.6) which seem to be the most biologically reasonable and the most well-studied in our specific context. First, we have the following proposition,

#### Proposition 4.8

For a circular genome with *n* oriented regions, the number of distinct rearrangement actions which apply 2 cuts (that is, the number of algebra elements ${\textbf{z}}\alpha {\textbf{z}}$ such that $|{{\,\mathrm{\textrm{CutPos}}\,}}(\alpha )| = 2$) is given by $\left\lfloor \frac{n}{2}\right\rfloor$.

#### Proof

Consider the possibilities for making two cuts. There are *n* possible positions to place a cut, and we want to choose two (this is why there are $$\left( {\begin{array}{c}n\\ 2\end{array}}\right) $$ cosets that achieve this). In the double coset case, some choices are equivalent due to dihedral symmetry of the regions, so we think about the distance between the two cuts (or equivalently, the sizes of the two segments). If *n* is even, we can choose two cuts that are at most $\frac{n}{2}$ regions apart. If *n* is odd, the cuts can be at most $\frac{n-1}{2}$ regions apart, and so the total number of options in each cases is $\left\lfloor \frac{n}{2}\right\rfloor$. Finally, for each choice of cuts there is only one non-trivial way to put the two segments back together, giving us $\left\lfloor \frac{n}{2}\right\rfloor$ total rearrangements requiring 2 cuts. $\square$

We can similarly consider the number of distinct rearrangement actions that require precisely 3 cuts. First, choose a configuration of the three cuts along the circular genome. That is, choose a partition of the number of regions *n* into 3 parts. Each partition leads to a different number of options for reconnecting the three segments. For example, if $n=6$, the partition $n\!=\!2\!+\!2\!+\!2$ (all parts are the same size) leads to two distinct events: inverting every segment, or inverting any two segments. The partition $n\!=\!1\!+\!1\!+\!4$ (two parts the same size) leads to three distinct events x: inverting every segment, inverting two segments of size 1, or inverting a segment of size 1 and the segment of size 4. In general, we have the following.

#### Lemma 4.9

After breaking a genome into three unlabelled segments, there are 2 distinct actions placing the genome back together again if the parts are all the same size, 3 distinct actions if two segments are the same size, and 4 distinct actions if the segments all have different sizes.$\square$

Lemma 4.9 can be obtained by considering the possible options for reconnecting parts of a genome, as shown in Fig. 5. Further, we can count the number of partitions of *n* with 0, 2 or 3 parts equal with the following lemma.

#### Lemma 4.10

There are $\left\lfloor \frac{n-1}{2}\right\rfloor$ partitions of *n* into 3 parts which have exactly 2 parts of equal size, and one partition into three equal parts if and only if 3|*n*. In other words, there are $1 - \lceil \frac{n}{3}\rceil + \left\lfloor \frac{n}{3}\right\rfloor$ partitions into 3 equal parts. $\square$

The number of integer partitions of *n* into exactly three parts is given by the sequence $\textrm{A001399}(n\!-\!3)$ (The O. E. I. S. Foundation 2020), and we can use this sequence along with the previous two lemmas to obtain the following closed-form expression.

#### Proposition 4.11

For a circular genome with *n* oriented regions, the number of distinct rearrangement actions which apply 3 cuts (that is, the number of algebra elements ${\textbf{z}}\alpha {\textbf{z}}$ such that $|{{\,\mathrm{\textrm{CutPos}}\,}}(\alpha )| = 3$) is given by,

$$\begin{aligned} \left\lfloor \frac{n^2}{3}\right\rfloor -\left\lfloor \frac{n}{2}\right\rfloor . \end{aligned}$$

#### Proof

Combining Lemma 4.9 and Lemma 4.10 we obtainFurther, the above can be simplified to obtain the required form via manipulating generating functions. $\square$

We currently have no expression for the number of rearrangement actions requiring four cuts, however we note that inversions and transpositions—which require just 2 or 3 cuts—are the most commonly considered rearrangement actions in the literature, and may be considered the most ‘biologically reasonable’ rearrangements to consider as single events (see Table 2). Adding rearrangements which require $>3$ cuts into a model, for example some block-interchanges (Huang et al. 2010), also requires additional considerations. For example, these rearrangements will be products of other elements in the model, potentially making assigning probabilities more difficult. Inverses must also be considered, since for inversions ${\textbf{z}}\alpha {\textbf{z}}$ requiring more than 3 cuts, the inverse $\alpha ^{-1}$ might not be a term in ${\textbf{z}}\alpha {\textbf{z}}$. In this case, ${\textbf{z}}\alpha ^{-1} {\textbf{z}}$ must be included in the model if one wishes to ensure that rearrangements can be ‘undone’ in a single step.

**Table 5:** The number of double cosets (i.e. distinct rearrangement actions) on *n* regions that require *k* cuts, for each *n* and *k*

			# ${\mathcal {D}}_n\alpha {\mathcal {D}}_n$ requiring *k* cuts						
n	$|{{\,\mathrm{{\mathcal {H}}}\,}}_n|$	#${\mathcal {D}}_n\alpha {\mathcal {D}}_n$	0	2	3	4	5	6	7
2	8	2	1	1					
3	48	4	1	1	2				
4	384	13	1	2	3	7			
5	3840	56	1	2	6	15	32		
6	46,080	381	1	3	9	41	114	213	
7	645,120	3486	1	3	13	70	342	1083	1974

### Breakpoints

In this section we will discuss breakpoints in the context of circular genomes in the position paradigm. Breakpoints have a number of different definitions in the literature, some of which are specific to particular symmetries or other assumptions (Sankoff and Trinh 2004; Fertin et al. 2009; Alexandrino et al. 2021). Broadly, breakpoints are the regions in the genome around which rearrangements have occurred. This definition is similar to the set of cuts which we defined in Definition 4.1. The main difference is that the set of cuts keeps track of the *positions* of the regions. While the specific position labels themselves are less important in the circular case (since positions can be rotated/flipped around without altering the genome) the cut set does describe a configuration of cuts around the genome. We will now provide a more formal definition of a breakpoint for circular genomes that is analogous to the set of cuts.

#### Definition 4.12

Let $\sigma$ be an oriented genome with *n* regions. The set of breakpoints of $\sigma$ is defined as,

$$\begin{aligned} \textrm{breakpoints}(\sigma ):= \left\{ (i, j) \in [n]^2 \ \Bigg \vert \begin{array}{l} \text {regions }\, i \,\text {and } \,j \,\text {are out of order with}\\ \text {respect to the reference} \end{array} \right\} . \end{aligned}$$

We can rewrite this definition more algebraically, as we did for the set of cuts required for a rearrangement in Definition 4.1. Let $\sigma$ be an oriented genome with *n* regions. The set of breakpoints can be written as,

$$\begin{aligned} \textrm{breakpoints}(\sigma )&= \left\{ (\sigma ^{-1}(i),\sigma ^{-1} c(i)) : i\in [n] \text { and } \sigma ^{-1} c(i) \ne c\sigma ^{-1}(i) \right\} \\&= \left\{ (\sigma ^{-1}(i),\sigma ^{-1} c(i)) : i\in [n] - \textrm{Fix}([c,\sigma ^{-1}])\right\} . \end{aligned}$$

We can see that the number of breakpoints for a genome $\sigma$ can be written

$$\begin{aligned} \textrm{bp}(\sigma ):= \left| \textrm{breakpoints}(\sigma )\right| = \frac{2n-|\textrm{Fix}([\sigma ^{-1},c])|}{2}. \end{aligned}$$

This is equivalent to a statement in a paper by Meidanis and Dias (2000), in which genomes are written in the content paradigm.

#### Proposition 4.13

For a genome $\rho$ written in the content paradigm, we have

$$\begin{aligned} \textrm{bp}(\sigma ) = \frac{2n-|\textrm{Fix}(r\rho r c)|}{2}. \end{aligned}$$

#### Proof

To show these are equivalent, note that a genome $\rho$ in the content paradigm can be written

$$\begin{aligned} \rho = \sigma ^{-1} c \sigma , \end{aligned}$$

where $\sigma$ represents the same genome represented by $\rho$, but in the position paradigm, as a map from regions to signed positions. We have,

$$\begin{aligned} \begin{aligned} r \rho r c&= r \sigma ^{-1} c \sigma r c \\&= r (\sigma ^{-1} c^{-1} \sigma )^{-1} r^{-1} c \quad \qquad {(\text{ since } r^{-1} = r)} \\&= ( r (\sigma ^{-1} c^{-1} \sigma ) r^{-1})^{-1} c \\&= \sigma ^{-1} c^{-1} \sigma c \quad \qquad \qquad \qquad \quad {(\text{ one } \text{ can } \text{ show } r (\sigma c^{-1} \sigma ^{-1}) r^{-1} = (\sigma c^{-1} \sigma ^{-1})^{-1})}\\&= [\sigma , c], \end{aligned} \end{aligned}$$

and it is easy to see that

$$\begin{aligned} |\textrm{Fix}([\sigma ^{-1},c])| = |\textrm{Fix}([\sigma ,c])|. \end{aligned}$$

Thus the two statements are equivalent. $\square$

## Discussion

While model-based approaches to estimating genome rearrangement distances can lead to significant computational challenges, similar approaches have been proven to be fruitful in other areas of phylogenetics. In particular, we contend that the development of these techniques will inevitably open up more possibilities for new distance estimates, lead to a deeper understanding of genome rearrangements, and allow us to compare and evaluate existing distances in a broader context.

The genome rearrangements literature covers a broad range of combinatorial approaches which have proven to be effective for a range of definitions of the genome rearrangement distance. However, these methods don’t yet benefit from the higher-level understanding that can be obtained from an algebraic perspective. Conversely, existing algebraic, model-based approaches could be improved by incorporating and unifying some of the knowledge contained within the combinatorial literature. We have reviewed existing literature, and provided examples and formulations which begin to strengthen this connection. With model-based approaches come the problems of identifying and defining sensible models, including possible rearrangements and associated probabilities. The ideas and examples we have presented here help to simplify this process.

There are a number of challenges which need to be overcome in order to further the development of algebraic, model-based approaches for computing rearrangement distances. The primary obstacle is the inherent computational complexity of these methods, due to the factorially large number of genomes. For example, as a distance, the maximum likelihood estimate of time taken to transform one genome into another incorporates all possible paths between genomes and can be computed under any rearrangement model without modification (Serdoz et al. 2017), however is extremely computationally intensive. The MLE distances can be made more tractable using representation theory (Terauds and Sumner 2018; Terauds et al. 2021; Terauds and Sumner 2022), and other related measures have similar benefits but are easier to compute, for example the mean first passage time (MFPT) (Francis and Wynn 2020; Terauds et al. 2021), which can also be computed under any rearrangement model. Such methods have so far not had the same level of attention in the literature as combinatorial approaches which focus on a single model of genome rearrangement, and there exists a wealth of knowledge of Markov chain theory and related concepts which may be applied in this area. We hope that formalising existing concepts from the literature will help to foster the development of new ideas, eventually allowing us to overcome some of the computational challenges inherent in genome rearrangement modelling problems.

## Data Availability

Data sharing is not applicable to this article as no datasets were generated or analysed during the current study.

## References

[CR1] Alexandrino A, Oliveira A, Dias U, Dias Z (2020). On the complexity of some variations of sorting by transpositions. JUCS J Univ Comput Sci.

[CR2] Alexandrino AO, Oliveira AR, Dias U, Dias Z (2021). Incorporating intergenic regions into reversal and transposition distances with indels. J Bioinform Comput Biol.

[CR3] Alexeev N, Aidagulov R, Alekseyev MA, Ortuño F, Rojas I (2015). A computational method for the rate estimation of evolutionary transpositions. Bioinform Biomed Eng.

[CR4] Bader David A, Moret Bernard ME, Mi Y (2001). A linear-time algorithm for computing inversion distance between signed permutations with an experimental study. J Comput Biol.

[CR5] Bafna V, Pevzner PA (1996). Genome rearrangements and sorting by reversals. SIAM J Comput.

[CR6] Bafna V, Pevzner PA (1998). Sorting by transpositions. SIAM J Discrete Math.

[CR7] Baudet C, Dias U, Dias Z (2015). Sorting by weighted inversions considering length and symmetry. BMC Bioinform.

[CR8] Berard S, Bergeron A, Chauve C, Paul C (2007). Perfect sorting by reversals is not always difficult. IEEE/ACM Trans Comput Biol Bioinf.

[CR9] Caprara A (1997) Sorting by reversals is difficult. In: Proceedings of the first annual international conference on computational molecular biology—RECOMB ’97. 10.1145/267521.267531

[CR10] Dalevi D, Eriksen N, Eriksson K, Andersson S (2002). Measuring genome divergence in bacteria: a case study using Chlamydian data. J Mol Evol.

[CR11] Darling Aaron E, István M, Ragan Mark A (2008). Dynamics of genome rearrangement in bacterial populations. PLoS Genet.

[CR12] Egri-Nagy A, Gebhardt V, Tanaka MM, Francis AR (2013). Group-theoretic models of the inversion process in bacterial genomes. J Math Biol.

[CR13] Feijao P, Meidanis J (2013). Extending the algebraic formalism for genome rearrangements to include linear chromosomes. IEEE/ACM Trans Comput Biol Bioinf.

[CR14] Felsenstein J (1973). Maximum likelihood and minimum-steps methods for estimating evolutionary trees from data on discrete characters. Syst Zool.

[CR15] Fertin G, Labarre A, Rusu I, Tannier E, Vialette S (2009). Combinatorics of genome rearrangements.

[CR16] Francis AR (2013). An algebraic view of bacterial genome evolution. J Math Biol.

[CR17] Francis AR, Wynn HP (2020). A mean first passage time genome rearrangement distance. J Math Biol.

[CR18] Galvao GR, Baudet C, Dias Z (2017). Sorting circular permutations by super short reversals. IEEE/ACM Trans Comput Biol Bioinform.

[CR19] Hannenhalli S, Pevzner P (1996). Transforming cabbage into turnip: polynomial algorithm for sorting signed permutations by reversals. J ACM.

[CR20] Helsgaun K (2009). General k-opt submoves for the Lin–Kernighan TSP heuristic. Math Program Comput.

[CR21] Huang Y-L, Huang C-C, Tang CY, Lu CL (2010). An improved algorithm for sorting by block-interchanges based on permutation groups. Inf Process Lett.

[CR22] Lefebvre J, El-Mabrouk N, Tillier E, Sankoff D (2003). Detection and validation of single gene inversions. Bioinformatics.

[CR23] Lin Yu, Moret Bernard ME (2008). Estimating true evolutionary distances under the DCJ model. Bioinformatics.

[CR24] Lin G-H, Xue G (2001). Signed genome rearrangement by reversals and transpositions: models and approximations. Theoret Comput Sci.

[CR25] Meidanis J, Dias Z (2000). An alternative algebraic formalism for genome rearrangements. Comp Genom.

[CR26] Oliveira AR, Fertin G, Dias U, Dias Z (2018). Sorting signed circular permutations by super short operations. Algorithms Mol Biol.

[CR27] Oliveira AR, Jean G, Fertin G, Dias U, Dias Z (2019). Super short operations on both gene order and intergenic sizes. Algorithms Mol Biol.

[CR28] Peng Q, Pevzner PA, Tesler G (2006). The fragile breakage versus random breakage models of chromosome evolution. PLoS Comput Biol.

[CR29] Pevzner P, Tesler G (2003). Human and mouse genomic sequences reveal extensive breakpoint reuse in Mammalian evolution. Proc Natl Acad Sci.

[CR30] Sangeeta B, Pedro F, Francis Andrew R (2018). Position and content paradigms in genome rearrangements: the wild and crazy world of permutations in genomics. Bull Math Biol.

[CR31] Sankoff D, Trinh P (2004) Chromosomal breakpoint re-use in the inference of geome sequence rearrangement. In: Proceedings of the eighth annual international conference on computational molecular biology—RECOMB-04. ACM Press, vol 8, pp 30–35. 10.1145/974614.974619

[CR32] Serdoz S, Egri-Nagy A, Sumner J, Holland BR, Jarvis PD, Tanaka MM, Francis AR (2017). Maximum likelihood estimates of pairwise rearrangement distances. J Theor Biol.

[CR33] Terauds V, Sumner J (2022). A new algebraic approach to genome rearrangement models. J Math Biol.

[CR34] Terauds V, Stevenson J, Sumner J (2021). A symmetry-inclusive algebraic approach to genome rearrangement. J Bioinform Comput Biol.

[CR35] Thanbichler M, Shapiro L (2006). Chromosome organization and segregation in bacteria. J Struct Biol.

[CR36] The O. E. I. S. Foundation (2020) The online encyclopedia of integer sequences. https://oeis.org/

[CR37] Venta T, Jeremy S (2018). Maximum likelihood estimates of rearrangement distance: implementing a representation-theoretic approach. Bull Math Biol.

[CR38] Walter MEMT, Sobrinho MC, Oliveira ETG, Soares LS, Oliveira AG, Martins TES, Fonseca TM (2005). Improving the algorithm of Bafna and Pevzner for the problem of sorting by transpositions: a practical approach. J Discrete Algorithms.

[CR39] Watterson GA, Ewens WJ, Hall TE, Morgan A (1982). The chromosome inversion problem. J Theor Biol.

